# Essential Developmental, Genomic Stability, and Tumour Suppressor Functions of the Mouse Orthologue of *hSSB1/NABP2*

**DOI:** 10.1371/journal.pgen.1003298

**Published:** 2013-02-07

**Authors:** Wei Shi, Amanda L. Bain, Bjoern Schwer, Fares Al-Ejeh, Corey Smith, Lee Wong, Hua Chai, Mariska S. Miranda, Uda Ho, Makoto Kawaguchi, Yutaka Miura, John W. Finnie, Meaghan Wall, Jörg Heierhorst, Carol Wicking, Kevin J. Spring, Frederick W. Alt, Kum Kum Khanna

**Affiliations:** 1 Queensland Institute of Medical Research, Herston, Australia; 2 School of Biomolecular and Physical Sciences, Griffith University, Nathan, Australia; 3 Howard Hughes Medical Institute, Immune Disease Institute, Program in Cellular and Molecular Medicine, Children's Hospital Boston, Boston, Massachusetts, United States of America; 4 Department of Genetics and Pediatrics, Harvard Medical School, Boston, Massachusetts, United States of America; 5 Department of Biochemistry and Molecular Biology, School of Biomedical Sciences, Monash University, Melbourne, Australia; 6 School of Medicine, University of Queensland, Herston, Australia; 7 Department of Bioregulation and Molecular Neurobiology, Nagoya City University, Graduate School of Medical Sciences, Nagoya, Japan; 8 SA Pathology, Institute of Medical and Veterinary Science, Adelaide, Australia; 9 Victorian Cancer Cytogenetics Service, St. Vincent's Hospital, Fitzroy, Melbourne, Australia; 10 Department of Medicine, St. Vincent's Hospital, Fitzroy, Australia; 11 St. Vincent's Institute, Fitzroy, Australia; 12 Institute for Molecular Bioscience, The University of Queensland, St. Lucia, Australia; St. Jude Children's Research Hospital, United States of America

## Abstract

Single-stranded DNA binding proteins (SSBs) regulate multiple DNA transactions, including replication, transcription, and repair. We recently identified SSB1 as a novel protein critical for the initiation of ATM signaling and DNA double-strand break repair by homologous recombination. Here we report that germline *Ssb1^−/−^* embryos die at birth from respiratory failure due to severe rib cage malformation and impaired alveolar development, coupled with additional skeletal defects. Unexpectedly, *Ssb1* ^−/−^ fibroblasts did not exhibit defects in Atm signaling or γ-H2ax focus kinetics in response to ionizing radiation (IR), and B-cell specific deletion of *Ssb1* did not affect class-switch recombination *in vitro*. However, conditional deletion of *Ssb1* in adult mice led to increased cancer susceptibility with broad tumour spectrum, impaired male fertility with testicular degeneration, and increased radiosensitivity and IR–induced chromosome breaks *in vivo*. Collectively, these results demonstrate essential roles of Ssb1 in embryogenesis, spermatogenesis, and genome stability *in vivo*.

## Author Summary

Single-stranded DNA binding proteins (SSBs) play a variety of roles in the cell, regulating transcription, replication, and DNA repair. We recently identified and described a novel SSB, designated SSB1, which was shown to be critical for DNA repair in the cell. In this study we have used a mouse model in which the *Ssb1* gene is deleted to further investigate its physiological function. Here, we show that deletion of *Ssb1* causes death at birth due to severe respiratory failure, which is caused by an improperly formed rib cage and immature lung development. In addition, we observed multiple additional skeletal defects in *Ssb1* deleted mice, indicating that Ssb1 is necessary for proper development of the embryonic skeleton. Furthermore, *Ssb1* deletion in the adult mouse caused fertility defects in male mice and led to the development of a variety of tumours. Together, these studies demonstrate a novel and critical role of Ssb1 in embryonic development, in fertility, and in the protection from tumour formation.

## Introduction

Appropriate and timely repair of damaged DNA is critical for maintaining genomic integrity and tumour suppression [1], [2]. DNA double-strand breaks (DSBs) are the most cytotoxic genomic lesions, and can arise from exogenous genotoxic insult, stalled replication forks, or during physiological processes such as meiosis and B and T cell maturation. Organisms have evolved two main pathways for DSB repair: non-homologous end joining (NHEJ) and homologous recombination (HR). In the initial step of HR, DSBs are resected to generate 3′ single-stranded DNA (ssDNA) tails. The ssDNA intermediates are protected from further degradation by ssDNA-binding proteins (SSBs).

The SSB family of proteins are conserved in all three kingdoms of life [3] and are characterised structurally by their oligonucleotide-binding (OB) folds that bind ssDNA. SSB proteins can be subdivided into two sub-groups. First, simple SSBs, typified by the *Escherichia coli* (*E. coli*) SSB, contain a single OB-fold. The second sub-group includes the higher ordered replication protein A (RPA), which contains multiple OB-folds and is conserved in yeast and higher eukaryotes [3]. Human RPA is a heterotrimeric polypeptide, widely believed to be a central component of both DNA replication and DNA repair pathways [4], [5], [6]. Recently, we identified two novel SSB proteins, named SSB1 (also known as OBFC2B, NABP2 or SSOS-B1) and SSB2 (also known as OBFC2A, NABP1 or SOSS-B2) [7], which are conserved in vertebrates but not in lower eukaryotes. These SSBs are more closely related to the bacterial and archaeal SSB sub-group than to RPA [3]. Both SSBs encode a conserved single OB-fold followed by a divergent spacer domain and a conserved C-terminal motif, suggesting functional overlap between these proteins. The spacer region is the only significant difference between human SSB1 and SSB2.

Our functional characterization of SSB1 revealed that it is stabilised following exposure of cells to ionizing radiation (IR) forming distinct foci at DSB sites [7]. Depletion of SSB1 compromises the DNA damage checkpoints and HR, resulting in an increased sensitivity to IR. Further studies showed that human SSB1 and SSB2 exist in two separate sub-complexes that also contain IntS3 and C9orf80 (also known as SSBIP1/MISE) [8], [9], [10], [11]. Similar to depletion of SSB1, silencing of INTS3 and C9orf80 results in defects in ATM signalling and HR as well as hypersensitivity to IR [8], [9], [10].

Here, we describe the generation of *Ssb1* knockout mice to define the physiological role of Ssb1. We report that germline deficiency for *Ssb1* causes perinatal lethality due to aberrant rib-cage formation and improper lung differentiation. Furthermore, conditional knockout of *Ssb1* in adult mice leads to reduced fertility in male mice, increased sensitivity to γ-irradiation and a predisposition to tumorigenesis. Taken together, our data demonstrate that Ssb1 is essential for embryogenesis and the maintenance of genomic stability *in vivo*.

## Results

### Ssb1 deficiency results in perinatal lethality

The murine *Ssb1* gene is located on chromosome 10 and spans 7 exons. We engineered a “floxed” *Ssb1* allele with unidirectional loxP sites flanking its major protein coding exons 3–6, including the OB-fold domain critical for its DNA binding activity (Figure S1A). Correct targeting was confirmed by Southern blot (Figure S1B) and genotyping PCR (Figure S1C). Evaluation of the growth of *Ssb1* heterozygous mice *(Ssb1^+/−^)* relative to wild-type littermates (*Ssb1^+/+^*) revealed no apparent physiological abnormalities in *Ssb1* ^+/−^ mice monitored for up to 2 years. To generate mice with targeted deletion of *Ssb1*, we intercrossed *Ssb1* ^+/−^ breeding pairs, with the expectation that approximately 25 percent of the offspring would be of an *Ssb1* ^−/−^ genotype. Interestingly, no viable *Ssb1* ^−/−^ mice were detected amongst more than one hundred offspring from these intercrosses genotyped at 12 days post-partum (Table I). These results suggested that *Ssb1* deletion might result in lethality during embryogenesis.

**Table 1:** Impact of deletion of *Ssb1* on embryonic survival in *Ssb1* ^+/−^ intercrosses.

		*Ssb1^+/+^*	*Ssb1^+/−^*	*Ssb1^−/−^*
E13.5 embryos	**Expected:**	*12*	*24*	*12*
*(48 genotyped)*	**Observed:**	*8*	*22*	*18*
E18.5 embryos	**Expected:**	*9*	*18*	*9*
*(36 genotyped)*	**Observed:**	*6*	*16*	*14*
Live Pups (P12)	**Expected:**	*32*	*64*	*32*
*(128 genotyped)*	**Observed:**	*39*	*89*	*0*

**Figure 1:**
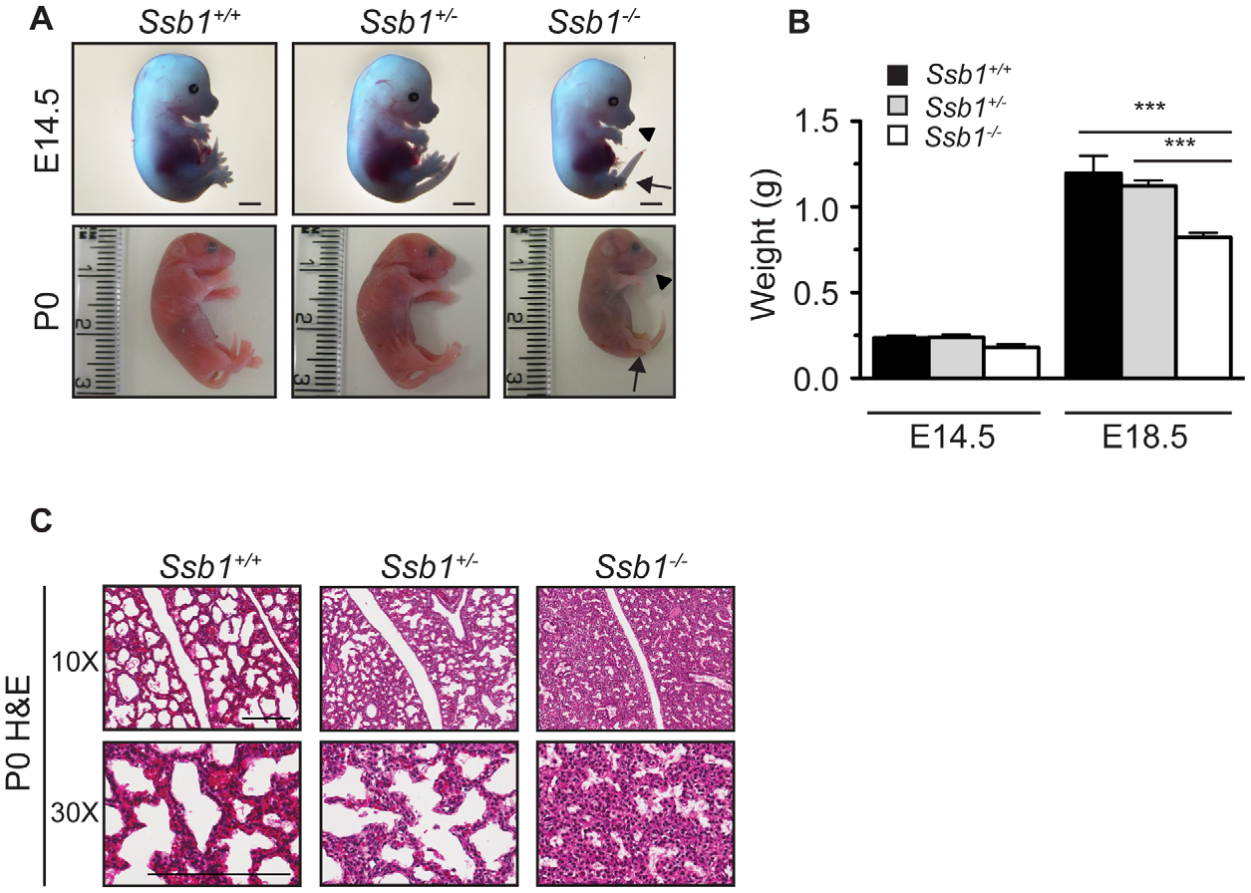
*Ssb1* deletion causes perinatal lethality due to severe respiratory failure. (A) Comparison of size and appearance of E14.5 and P0 *Ssb1^+/+^*, *Ssb1^+/−^*, and *Ssb1* ^−/−^ offspring. Note the cyanosis in P0 *Ssb1* ^−/−^ pups. *Scale = 2 mm*. (B) Comparison of weights of E14.5 and E18.5 *Ssb1^+/+^*, *Ssb1 ^+/−^* and *Ssb1^−/−^* embryos. Data represent the mean ± SEM, *n* = 3–16 embryos per group from a minimum of 3 litters per timepoint (***P<0.001; student's *t*-test). (C) Haematoxylin and eosin staining of P0 lungs of *Ssb1* ^+/+^, *Ssb1* ^+/−^ and Ssb1^−/−^ pups delivered by caesarian section.

**Figure 2:**
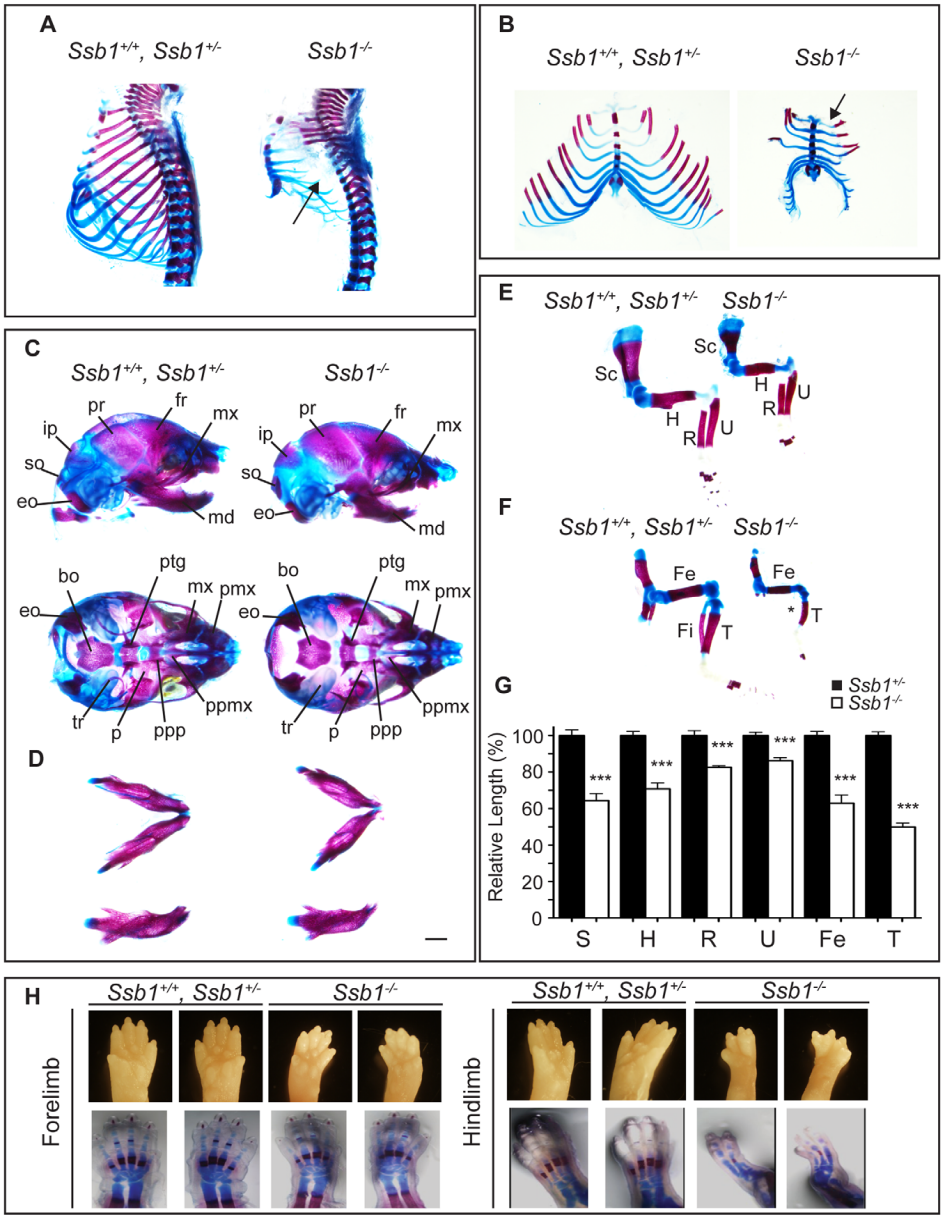
*Ssb1* deletion causes multiple skeletal defects. (A) Alcian blue and Alizarin red staining of control (*Ssb1* ^+/+^, *Ssb1* ^+/−^) and *Ssb1* ^−/−^ E18.5 ribcages. (B) Butterfly rib-spread of *Ssb1* control and *Ssb1* ^−/−^ E18.5 ribcages. (C) Comparison of skull anatomy of *Ssb1* control and *Ssb1* ^−/−^ E18.5 embryos. eo, exoccipital; so, supraoccipital; ip interparietal; pr, parietal; fr, frontal; mx, maxilla; md, mandible; bo, basioccipital; ptg, pterygoid; pmx, premaxilla; ppmx, palatal process maxilla; ppp, palatal process palatine; p, palatine; tr, tympanic ring. (D) Comparison of *Ssb1* control and *Ssb1* ^−/−^ mandibles *(Scale = 1 mm)*. (E) Forelimb and (F) Hindlimb of E18.5 *Ssb1* control and *Ssb1* ^−/−^ embryos. Sc, scapula; H, humerus; R, radius; U, ulna; F, femur; T, tibia. * designates missing fibula. (G) Quantification of long bone measurements of the forelimb and hindlimb of *Ssb1* ^+/−^ and *Ssb1* ^−/−^ E18.5 limbs. Data represent the mean ± SEM of bone length (*n* = 3 per condition, ***P<0.001, student's *t*-test). (H) Whole-autopod (top) and skeletal preparation (bottom) of E18.5 forelimbs (left) and hindlimbs (right) from *Ssb1* control and *Ssb1* ^−/−^ embryos.

In order to define the time point of embryonic lethality caused by Ssb1 ablation, we collected embryos from *Ssb1* ^+/−^ intercrosses at different gestational days, assessed by the presence of a vaginal plug at E0.5. *Ssb1* ^−/−^ embryos were recovered at near-Mendelian ratios at E13.5 and E18.5 (Table I), but were significantly growth retarded in terms of both body weight and length at the latter time-point, when compared to wild-type and heterozygous littermates (Figure 1A, 1B; Figure S2A, S2B). *Ssb1* ^+/+^ and *Ssb1* ^+/−^ embryos were morphologically indistinguishable, in terms of both body size and body length. *Ssb1^−/−^* embryos also displayed craniofacial abnormalities, including a recessed mandible (lower jaw) and misshapen snout (Figure 1A, arrowheads; Figure 2C, 2D, Figure S2C). Furthermore, there was a defect in the outgrowth of both fore- and hindlimbs, as well as hindlimb-specific oligodactyly (missing digits) (Figure 1A, arrows). However, these embryos appeared otherwise grossly normal, suggesting that Ssb1 ablation may cause lethality during the perinatal period.

To further investigate the cause of *Ssb1^−/−^* lethality, we performed caesarian recovery of embryos at E18.5 or at the time of birth (P0), and stimulated breathing by clearing the facial orifices and gentle stroking of the snout. In the litters examined, all *Ssb1^+/+^* and *Ssb1^+/−^* pups established rhythmic breathing, a healthy pink skin color and movement within minutes. However, *Ssb1^−/−^* pups rapidly became asphyxic and typically died between 10∼30 min post caesarian excision, despite taking short, sporadic gasping breaths, suggesting that they could not breathe and oxygenate their blood properly (Figure 1A, Figure S2A). Haematoxylin and eosin (H&E) staining on these embryos suggested that atelectasis was the primary cause of respiratory failure (Figure 1C). These results suggest that *Ssb1* ^−/−^ embryos survive the entire course of development *in utero* but die at the perinatal stage.

### Ssb1 ablation results in aberrant skeletal patterning

To further investigate the abnormalities we observed in the craniofacial region and hindlimb of *Ssb1* ^−/−^ embryos, we next sought to determine if their skeletal architecture was altered by performing whole-mount cartilage and mineralized bone staining with alcian blue and alizarin red. Strikingly, we observed a number of defects in formation of both the axial and appendicular skeleton. Most notably, the ribcage of *Ssb1* ^−/−^ embryos was poorly formed, small in size, and exhibited an almost complete lack of ossification when compared to control littermates (*Ssb1* ^+/+^, *Ssb1* ^+/−^) (Figure 2A). This defect led to the appearance of “floating ribs”, with no evidence of ossification in all but the four most anterior rib pairs. In addition, the ribcage of *Ssb1* ^−/−^ embryos was misshapen, with a lack of curvature in the anterior ribs, and horizontally orientated rib-sternum attachments (Figure 2A, Figure 2B, arrow). The more posterior “floating” ribs in these embryos were also rudimentary and abnormally shaped, contributing to a general decrease in size of the rib-cage (Figure 2A, Figure 2B). The lack of structural support from the misshapen and poorly developed rib-cage in *Ssb1* ^−/−^ embryos would have significantly contributed to the respiratory distress evident in these embryos at birth, and resulted in rapid atelectasis and perinatal death.

Examination of the skull of E18.5 embryos revealed normal formation of major bone structures, including the parietal (pr), intraparietal (ip), frontal (fr) and supraoccipital (so) bones. We noted a modest elongation of the premaxillary bone (pmx), consistent with the pointed snout seen in these embryos, and a shortened mandible (micrognathia), which was set at a wider angle than in control embryos (*Ssb1* ^+/+^, *Ssb1* ^+/−^) (Figure 2C, 2D). The tympanic ring (tr), which supports the eardrum, was also poorly formed in *Ssb1* ^−/−^ embryos (Figure 2C). Furthermore, we observed evidence of a variably penetrant cleft palate (*n = *2 of 5 embryos), which was evident even between *Ssb1* ^−/−^ mice of the same litter (Figure S2D–S2F; arrows, arrowheads). Together, these data suggest a spectrum of craniofacial abnormalities in *Ssb1* ^−/−^ embryos.

The limb skeleton of *Ssb1* ^−/−^ E18.5 embryos showed a significant decrease in the length of all long bones, including humerus, radius, ulna, femur and tibia, as well as the scapula (Figure 2E–2G), indicating a limb outgrowth defect (***P<0.001, Figure 2G). Overall, this phenotype was more pronounced in the hindlimbs, where we observed varying degrees of abnormalities in these structures, including absent fibulas (Figure 2F). Finally, although the forelimbs of *Ssb1* ^−/−^ embryos were properly patterned (albeit smaller in size), hindlimbs displayed aberrant bone mineralization and severe defects in patterning along the anterior-posterior axis, which always manifested as oligodactyly (Figure 2H). Interestingly, this phenotype was variable in penetrance, with between two to a maximum of four digits present, and we often observed variation of patterning defects between the left and right hindlimb autopods within the one embryo. Taken together, these data indicate that Ssb1 is necessary for skeletogenesis and hindlimb digit specification in the embryo, and that it is of particular importance for the later steps of chondrogenesis involving bone ossification. These data highlight a novel and unexpected role for Ssb1 during embryogenesis.

### *Ssb1* ^−/−^ embryos exhibit distal lung differentiation defects

**Figure 3:**
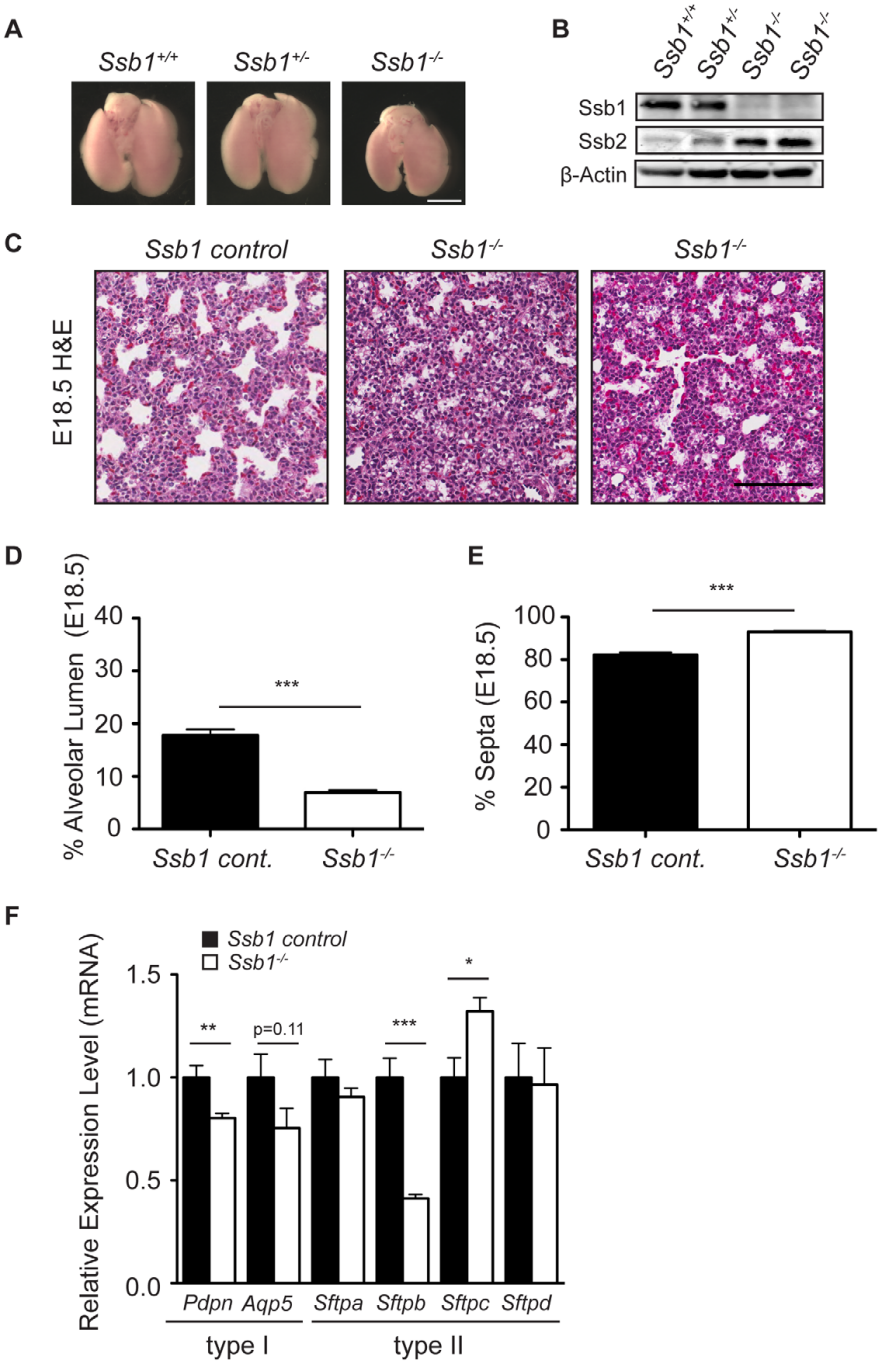
Distal lung differentiation defects in *Ssb1* ^−/−^ embryos. (A) Representative image showing the comparison of lung size and morphology of E18.5 *Ssb1* ^+/+^, *Ssb1* ^+/−^ and *Ssb1* ^−/−^ embryos. *Scale bar = 2 mm*. (B) Protein level of Ssb1, Ssb2 and Actin in *Ssb1* ^+/+^, *Ssb1* ^+/−^ and *Ssb1* ^−/−^ E18.5 lungs. (C) Haematoxylin and eosin staining showing morphology of alveolar lumen formation and intra-alveolar septae thickness in *Ssb1* control (*Ssb1* ^+/+^, *Ssb1* ^+/−^) and *Ssb1* ^−/−^ E18.5 embryos. (D) Quantitation of alveolar lumen area and (E) intra-alveolar septa area in *Ssb1* control (*Ssb1* ^+/+^, *Ssb1* ^+/−^) and *Ssb1* ^−/−^ embryos. Data represent the mean ± SEM (*n* = 3 control and 4 *Ssb1* ^−/−^ embryos from 2 litters. ***P<0.001, student's *t*-test). (F) qRT-PCR of Type I and Type II alveolar epithelial cell markers in *Ssb1* control (*Ssb1* ^+/+^, *Ssb1* ^+/−^) and *Ssb1* ^−/−^ E18.5 lungs. Data represent mean ± SEM from 4 embryos per condition (*P<0.05, **P<0.01,***P<0.001; student's *t*-test).

To determine if other causative factors may have contributed to the perinatal lethality in *Ssb1^−/−^* embryos, we next performed histological analysis of sagittal sections from E18.5 embryos. We observed grossly normal morphology for major organs including the brain, heart, thymus, intestine, and liver (Figure S3). However, consistent with the respiratory distress phenotype, we observed immature lung morphology in these sections (Figure S3). To more closely examine this, we dissected lungs from E18.5 *Ssb1^+/+^*, *Ssb1^+/−^* and *Ssb1^−/−^* embryos (Figure 3A) and confirmed complete deletion of the Ssb1 protein by western blot (Figure 3B). Interestingly, we also noted an increase in the protein level of Ssb2 in *Ssb1* ^−/−^ lungs (Figure 3B), similar to what has been observed based on siRNA depletion in human cells [8], [9], [10]. A comparison of the gross morphology of the lungs revealed that the lungs of *Ssb1* ^−/−^ embryos were consistently smaller than their *Ssb1* ^+/+^ and *Ssb1* ^+/−^ counterparts when measured in terms of lobe length and width (Figure 3A, data not shown), although this was in proportion to the overall growth retardation in these embryos. In addition, lungs of *Ssb1* ^−/−^ embryos were correctly lobulated, with four right lobes and a single left lobe flanking the heart, suggesting that early lung development patterning in these embryos is intact (Figure 3A). However, H&E analysis on coronal sections of these lungs revealed aberrant late-stage lung development, with reduced alveolar lumens and thickened, hypercellular alveolar walls in *Ssb1* ^−/−^ lungs when compared to control (*Ssb1* ^+/−^ and *Ssb1* ^+/−^) littermates (Figure 3C–3E ***P<0.001). During lung development, regression of the mesenchyme occurs from approximately E15.5 onwards by apoptosis to form the air-blood barrier, necessary for efficient respiration. To determine if the higher cell density in *Ssb1* ^−/−^ lungs results from either a decrease in apoptosis during development or increased proliferation, we performed immunohistological staining on E14.5 and E18.5 lung sections for ApopTag and Ki67, respectively. However, no differences in the levels of Ki67 or ApopTag were observed at these developmental stages (Figure S4).

Perinatal death due to respiratory failure can be caused by impaired differentiation of the proximal and/or distal airway epithelium. To determine if proximal airway epithelium was properly differentiated, we examined levels of *Cc10* (also known as *Scgb1a1/Ccsp*), a marker for secretory Clara cells, as well as *Foxj1* (also known as *Hfh-4*), a marker of ciliated epithelial cells in the proximal epithelium by quantitative real-time PCR (qPCR) in *Ssb1* control (*Ssb1* ^+/+^, *Ssb1* ^+/−^, *n = *4) and *Ssb1* ^−/−^ (*n = *4) lung tissue at E18.5. In addition, we also examined transcript levels of Cd31, a marker of endothelial cells. These analyses revealed no significant differences in the mRNA levels of these markers, suggesting that both proximal airway differentiation and blood vessel formation of *Ssb1* ^−/−^ lungs are intact (Figure S5A). Furthermore, immunohistological staining of smooth muscle actin revealed normal bronchi and bronchioli development in these embryos (Figure S5B). Next, we examined differentiation of the distal saccules which contain alveolar epithelial type I and type II cells (AECs), responsible for gas exchange and the maintenance of surface tension through surfactant protein secretion, respectively. To determine if *Ssb1* ^−/−^ embryos exhibited defective differentiation in either of these cell types, we performed qPCR on E18.5 control and *Ssb1* ^−/−^ embryos to assess the transcript levels of *Aqp5* and *Pdpn*, as markers of type I AECs, as well as the surfactant protein transcripts *Sftpa*, *Sftpb*, *Sftpc* and *Sftpd*, as markers of type II AECs. Although *Sftpa* and *Sftpd* were unaffected by Ssb1 ablation, we observed a −2.4 fold change in *Sftpb* expression, suggesting aberrant type II AEC differentiation (***P<0.001, Figure 3F). Notably, deletion of *Sftpb* in the mouse has been shown to result in severe neonatal respiratory distress syndrome, and is the only surfactant protein that is indispensable for neonatal survival [12], [13], [14]. In addition to the decrease in *Sftpb* levels, we also observed a smaller (−1.25 fold) change in *Pdpn*, a type I AEC marker (**P<0.01), as well as small, but statistically non-significant decrease in *Aqp5*, another type I AEC marker (Figure 3F). As type II cells are thought to trans-differentiate to type I cells, this may be a secondary effect of improper type II AEC differentiation [15], [16]. Interestingly, we also observed a 1.3 fold increase in *Sftpc* mRNA (*P = 0.01, Figure 3F). As pro-SPC is expressed from E11.5 to E17.5 in lung epithelial progenitor cells, the relative increase in this transcript may simply represent developmental immaturity of *Ssb1* ^−/−^ lungs [16]. This is in accordance with blinded assessment by an independent pathologist, who observed an increase of immature type II AECs in the lungs of *Ssb1* ^−/−^ P0 embryos. These data indicate that Ssb1 is necessary for proper lung differentiation in the late stages of embryogenesis. Taken together, our results point to an important and novel role of Ssb1 in skeletal and lung differentiation.

### Ssb1 is not required for the response to DNA double-strand breaks in mouse embryonic fibroblasts or during class switch recombination

Mouse embryonic fibroblasts (MEFs) from *Ssb1* ^+/+^ and *Ssb1* ^−/−^ E13.5 embryos were isolated to investigate the role of Ssb1 in DSB repair and signaling in the mouse. Early passage *Ssb1* ^+/+^ and *Ssb1* ^−/−^ MEFs exhibited similar cell-cycle profiles, but *Ssb1* ^−/−^ MEFs had a slightly diminished proliferative capacity and more rapidly reached the plateau phase when compared with *Ssb1* ^+/+^ MEFs (Figure S6A, S6B). As we and others had previously described a role of SSB1 in the activation of ATM signaling in response to IR based on siRNA depletion in human cells [7], [9], [10], we assessed activation of this pathway in MEFs. Although we observed stabilization of Ssb1 in response to IR, interestingly, no attenuation of Atm activation was detected when we assessed autophosphorylation of Atm on serine1987 (serine1981 in human) or phosphorylation of its downstream activation target p53 on serine18 (serine15 in human) (Figure S6C). Similar to what we observed in *Ssb1* ^−/−^ lungs, Ssb2 protein levels were upregulated in *Ssb1* ^−/−^ MEFs. These results suggest that deletion of Ssb1 does not abrogate Atm activation in MEFs, and may highlight potential redundancy between Ssb1 and Ssb2 in these cells. To determine if the response to ionizing radiation was intact in *Ssb1* ^−/−^ cells, we also assessed the dynamics of γ-H2ax foci formation in *Ssb1* ^+/+^ and *Ssb1* ^−/−^ MEFs by immunofluorescence. These studies revealed no significant differences in the baseline level of γ-H2ax foci induction nor in the clearance of IR-induced γ-H2ax foci (Figure S6D, S6E), indicating that these cells did not exhibit higher levels of endogenous DNA damage and/or defective repair of IR-induced DSBs.

Next, we sought to utilise an *in vivo* model of DSB repair to interrogate if Ssb1 is necessary for DSB repair in the mouse. Class switch recombination (CSR) involves a programmed Ig heavy gene rearrangement in B-lymphocytes that requires repair of physiological DSBs generated as a result of activation-induced deaminase (AID) catalysed DNA base damage. In B-lymphocytes, the initial secreted antibodies contain heavy chains of the IgM class (or IgD formed via alternative splicing). Upon stimulation of these B-lymphocytes by antigen, the original IgM class heavy chain gene undergoes CSR to encode heavy chains of IgG, IgE, or IgA classes [17]. Several proteins involved in DSB repair including ATM, H2AX and 53BP1 have been suggested to have a role in CSR, to different extents, probably due to their roles in synapsis and/or DNA repair [17].

**Figure 4:**
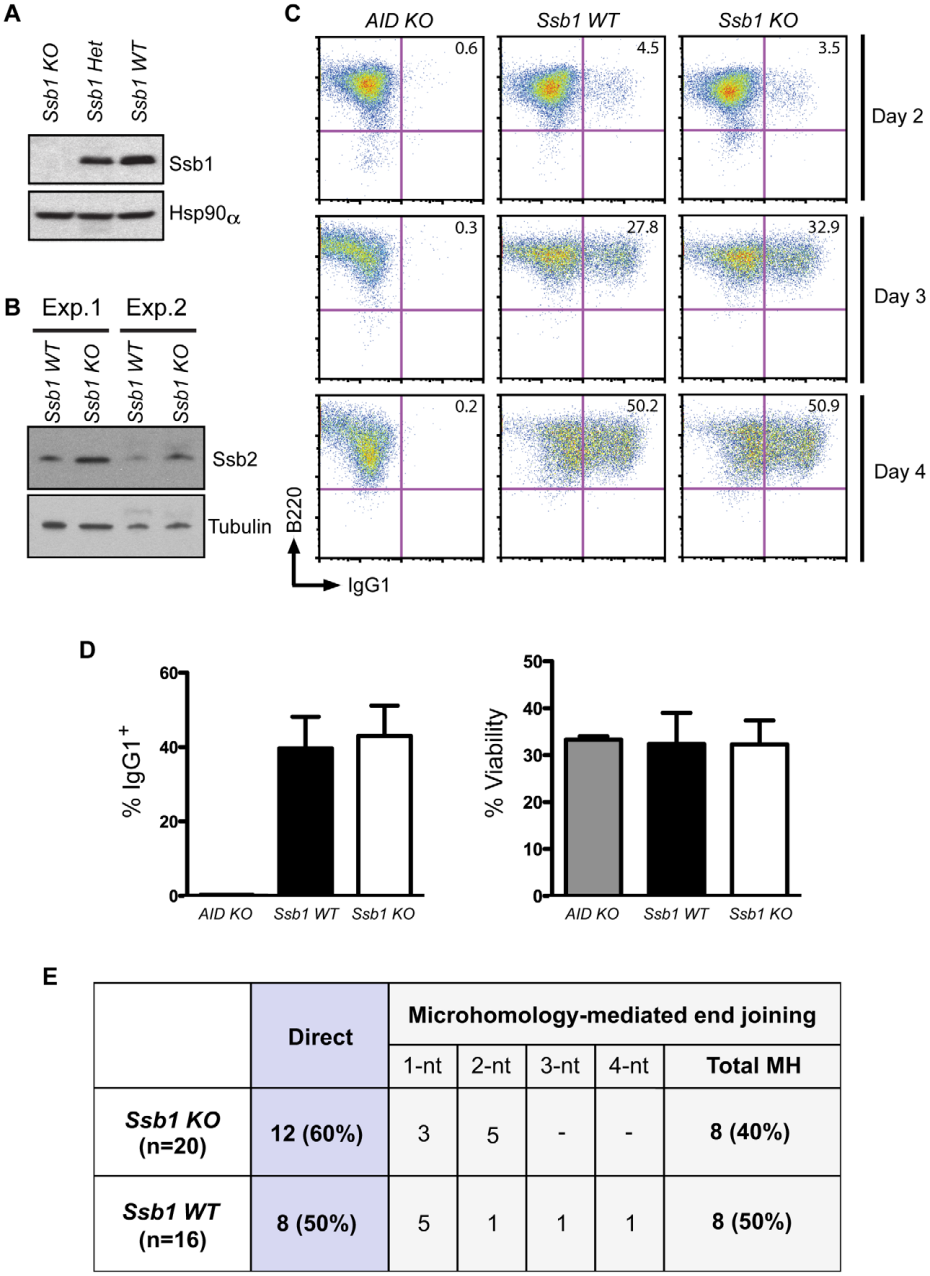
Class switch recombination activity in B-cell specific *Ssb1*-deleted mice. *Ssb1 ^fl/fl^* mice were crossed with a *Cd19*-Cre transgene expressing C57BL/6J mice to specifically delete *Ssb1* in B cells. Splenic B cells were isolated and stimulated for 2, 3, and 4 days using anti-Cd40 antibodies plus IL-4 to induce CSR to IgG1. (A) Western blotting of whole cell extracts showed loss of Ssb1 protein in stimulated B cells from *Ssb1* knockout (*Ssb1* KO; *Cd19*Cre^+^: *Ssb1^−/−^*) mice. Equal amounts of stimulated B cell extracts from heterozygous *Ssb1* (*Ssb1* Het; *Cd19*Cre^+^: *Ssb1^+/−^*) and wild-type *Ssb1* (*Ssb1* WT; *Cd19*Cre^+^: *Ssb1^+/+^*) mice were included for comparison. Equal loading was confirmed by probing for Hsp90α. (B) Western blotting of Ssb2 levels in B cells from *Ssb1* WT and *Ssb1* KO mice. (C) FACS analysis of CSR to IgG1 over time in stimulated B cells from mice of the indicated genotypes. Stimulated splenic B cells from *AID^−/−^* (*AID* KO) mice served as a negative control. (D) Summary statistics of CSR activity to IgG1 and viability on day 3 of stimulation. Mean and S.E.M. from three independent experiments are shown. No statistically significant differences (two-tailed unpaired *t*-test) were found. (E) Switch region junction analysis. Sm-Sg1 junctions were amplified from IL4 plus anti-CD40 stimulated primary B cells (day 4) and sequenced. Percentage and total numbers of direct or microhomology-mediated joints are indicated. nt, nucleotides.

To assess whether loss of Ssb1 affects CSR, we generated B cell specific conditional *Cd19*-Cre^+^: *Ssb1^−/−^* mice. Western blotting of whole cell extracts showed loss of Ssb1 protein in B cells from *Cd19*-Cre^+^: *Ssb1^−/−^* mice (Figure 4A) and upregulation of Ssb2 protein levels (Figure 4B), similar to what we observed in *Ssb1* ^−/−^ lungs and MEFs (Figure 3B, Figure S6C). Mice lacking Ssb1 in the B lineage produced normal numbers of mature IgM^+^ lymphocytes in the bone marrow and had spleens of normal size and cellularity. Upon *in vitro* stimulation of B cells isolated from spleens with anti-CD40 antibody plus IL-4 over 3 days, the extent of IgM to IgG1 switching and cell viability in wild-type and Ssb1-deficient B cells was also comparable (Figure 4C, 4D). No difference was found in the percentage and total numbers of direct or microhomology-mediated joins in switch region junctions from IL4 plus anti-CD40 stimulated primary *Ssb1* ^−/−^ B cells and wild-type B cells (Figure 4E). These results suggest that Ssb1 is dispensable for DSB repair by class-switch recombination.

### Conditional *Ssb1* gene deletion in adult mice

Given the perinatal lethality we observed in constitutive *Ssb1* ^−/−^ mice, we next employed a conditional approach to ubiquitously ablate Ssb1 postnatally using a tamoxifen-inducible Cre system by interbreeding *Ssb1^fl/fl^* mice with the *Rosa26*-CreER^T2^ strain (Figure S7) [18]. Efficiency of *Ssb1* deletion in adult mice (4 weeks old) following a series of tamoxifen injections was confirmed by both PCR for genomic recombination, and western blot analysis for protein depletion in various tissues (Figure S8A–S8C). The floxed *Ssb1* allele was efficiently deleted in bone marrow (BM), thymus, spleen, testes and small intestine, partially deleted in lung, kidney, liver and heart, but not in the brain (Figure S8A). Dramatically decreased Ssb1 protein levels were confirmed in multiple tissue samples from tamoxifen induced *Rosa26*-CreER^T2^: *Ssb1* ^−/−^ mice, with undetectable levels of Ssb1 protein in splenocytes and thymocytes as early as 10 days after the final tamoxifen induction (Figure S8B). Interestingly, we observed a dramatic up-regulation of Ssb2 in response to Ssb1 ablation in the bone marrow and spleen, but not in the testes and thymus of *Rosa26*-CreER^T2^: *Ssb1* ^−/−^ mice (Figure S8C).

### Impaired fertility in conditional *Rosa26*-CreER^T2^: *Ssb1* ^−/−^ male mice

**Figure 5:**
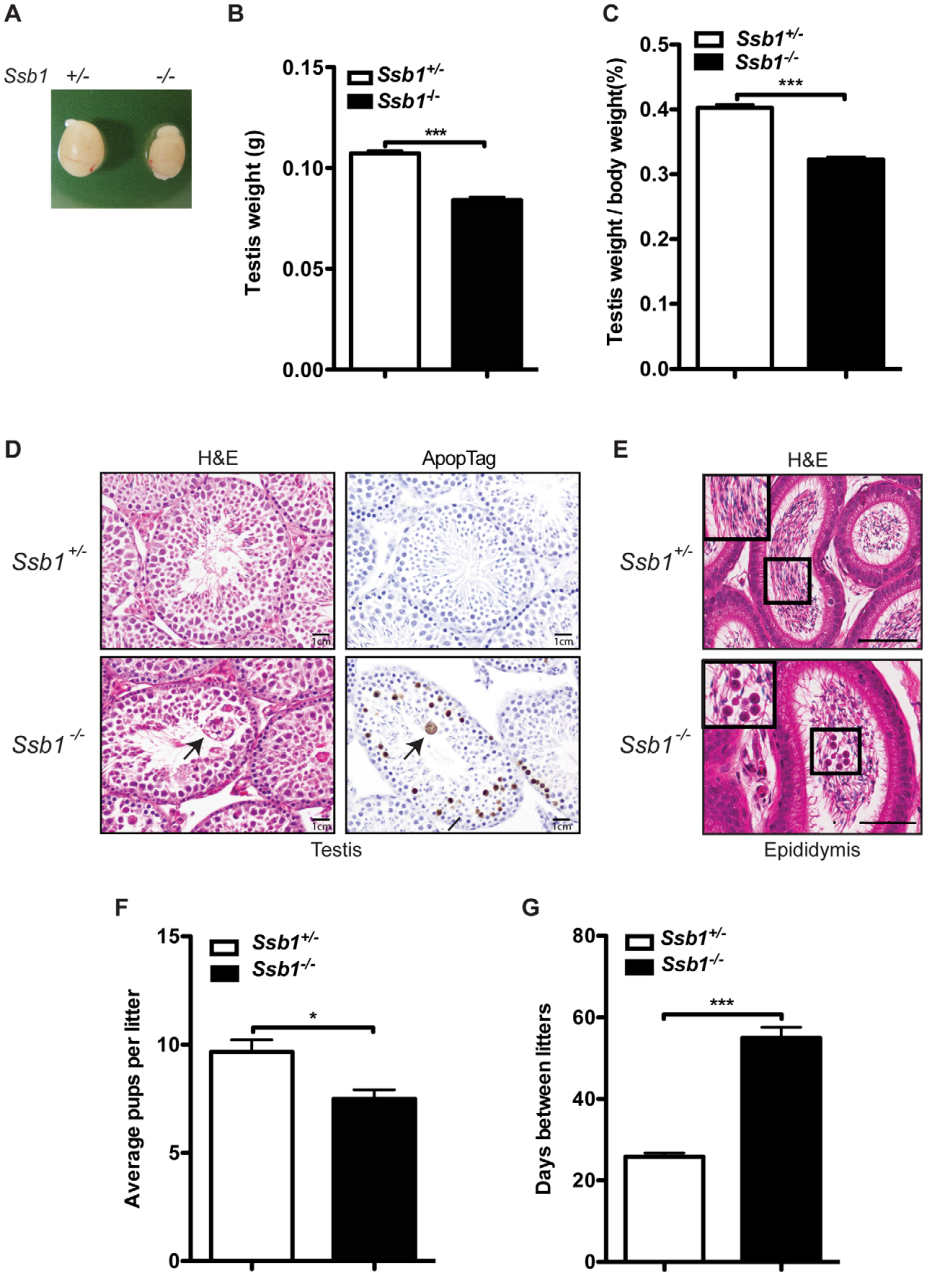
Testicular degeneration and impaired fertility in conditional *Rosa26*-CreER^T2^: *Ssb1* ^−/−^ male mice. (A) Representative image of testes from Rosa26-CreER^T2^: *Ssb1* ^−/−^ mice compared with those from *Rosa26*-CreER^T2^: *Ssb1^+/−^* littermates at 10 weeks of age. (B) Testis weight and (C) the gonado-somatic index (GSI) in conditional *Ssb1* deleted male mice compared with their littermates. Data represents the mean ± SEM of testis weight (*n* = 8, ***P<0.001; student's *t-*test) and the GSI (C) from 8 mice in each group. (D) Representative images of testis sections stained with Haematoxylin and eosin (left panels) and ApopTag for the detection of apoptotic cells (right panels). Note the multinucleated giant cells (black arrow) frequently present in the lumen in the testes from *Rosa26*-CreER^T2^: *Ssb1* ^−/−^ mice. Spermatogenic cells in *Rosa26*-CreER^T2^: *Ssb1* ^−/−^ testes showed an elevated apoptotic marker (black arrows, lower panel, right, *Scale = 25 µm*). (E) Haematoxylin and eosin staining showing epididymides of *Rosa26*-CreER^T2^: *Ssb1* ^−/−^ mice containing prematurely sloughed developing germ cells. The upper panel displays maturing spermatozoa in the wild type epididymis. In contrast, immature germ cells (black arrow) are present in the epididymis of *Rosa26*-CreER^T2^: *Ssb1* ^−/−^ mice (lower panel). Embedded images in the left corners show magnified views of the selected areas *(Scale = 50 µm*). (F) Litter size from *Rosa26*-CreER^T2^: *Ssb1* ^−/−^ male breeders (*n* = 4) with wild type female mice compared with those from *Rosa26*-CreERT2: *Ssb1^+/−^* breeders (*n* = 6, *P<0.05; student's *t-*test). (G) Litter interval in *Rosa26*-CreER^T2^: *Ssb1* ^−/−^ male mice (average of 63 days, *n* = 4) and Rosa26-CreER^T2^: *Ssb1^+/−^* males (average of 27 days, *n* = 6, ***P<0.001; student's *t-*test).

Monitoring of *Rosa26*-CreER^T2^: *Ssb1* ^−/−^ mice and control *Rosa26*-CreER^T2^: *Ssb1* ^+/−^ mice revealed no significant differences in body weight over a period of up to 90 weeks (Figure S9). In addition, histological analysis of all major organs, including the brain, thymus, lung, heart, liver, kidney and small intestine revealed no gross abnormalities. The abrogation of many DNA repair factors, (such as Atm [19], H2ax [20], Mdc1 [21] and Mcph1/Brit1 [22]) has been shown to result in impaired fertility, due to important roles of these proteins in DSB repair during meiosis. To determine the impact of Ssb1 deficiency on fertility, we examined ovaries and testes of *Rosa26*-CreER^T2^: *Ssb1* ^−/−^ mice six weeks after induction with tamoxifen. Whereas *Rosa26*-CreER^T2^: *Ssb1* ^−/−^ ovaries were morphologically normal in females, the testes from *Rosa26*-CreER^T2^: *Ssb1* ^−/−^ males were reduced in size (Figure 5A), in terms of both absolute weight (*n* = 8, ***P<0.001, Figure 5B) and gonado-somatic index (GSI) [23], an indicator of gonad weight as a proportion of total body mass (*n* = 8, ***P<0.001, Figure 5C), when compared to their *Rosa26*-CreER^T2^: *Ssb1* ^+/−^ littermates.

Histological examination of testes from 3-month-old *Rosa26*-CreER^T2^: *Ssb1* ^−/−^ male mice showed bilateral testicular degeneration with a spectrum of alterations in spermatogenesis. Testicular tubules showed degenerate, sometimes vacuolated, or necrotic spermatogenic cells, the latter with pyknotic nuclei and hypereosinophilic cytoplasm, or apoptotic body formation. Multinucleated giant cells were also frequently present in the lumen, either derived from spermatocytes with arrested development or the coalescence of spermatids (Figure 5D, left panel). Increased apoptosis at approximately the same stage, equivalent to stage IV of the normal seminiferous epithelial cycle has been reported in a number of mutants defective for meiotic recombination and/or meiosis-specific chromosome structures [24]. We performed ApopTag staining to determine the rate of spermatocyte apoptosis in testes from *Rosa26*-CreER^T2^: *Ssb1* ^+/*−*^ and *Rosa26*-CreER^T2^: *Ssb1* ^−/−^ littermates. As expected, the spermatocytes of *Rosa26*-CreER^T2^: *Ssb1* ^−/*−*^ testes exhibited increased ApopTag staining, compared to *Rosa26-*CreER^T2^: *Ssb1* ^+/*−*^ spermatocytes that were uniformly immunonegative for apoptosis (Figure 5D, right panel). As newly formed spermatozoa are released for passage into the epididymis for further maturation, we examined epididymides from *Rosa26*-CreER^T2^: *Ssb1* ^−/−^ mice for developing germ cells that were prematurely sloughed from the seminiferous epithelium and passed into the epididymis. The presence of round germ cells within the lumen of the epididymis (Figure 5E) suggests that, in addition to apoptosis, a significant number of germ cells were being lost via premature sloughing from the supporting Sertoli cells. Taken together, these results reveal a spectrum of testicular degenerations in the *Rosa26*-CreER^T2^: *Ssb1* ^−/−^ mice.

To further characterize the consequences of Ssb1 ablation on fertility, we interbred induced *Rosa26*-CreER^T2^: *Ssb1* ^−/−^ mice with wild-type mice. Consistent with the normal physiological appearance of their ovaries, induced female *Rosa26*-CreER^T2^: *Ssb1^−/−^* mice at ten weeks of age were found to be fertile. In contrast, only 4 out of 6 pairings of male *Rosa26*-CreER^T2^: *Ssb1* ^−/−^ mice with wild-type females led to successful pregnancies. In addition, in the 4 successful breeding pairs, we observed significantly smaller litter sizes (*P<0.05, Figure 5F) and much longer litter intervals (63 days vs. 27 days, ***P<0.001, Figure 5G) for *Rosa26*-CreER^T2^: *Ssb1* ^−/−^ breeders compared to *Rosa26*-CreER^T2^: *Ssb1* ^+/−^ control males. Histological analysis of testes sections revealed a dramatically decreased number of elongated spermatids in the infertile compared to the fertile *Ssb1*-deleters. Thus, post-natal *Ssb1* deletion leads to a spectrum of partial to complete male fertility defects, demonstrating the importance of this protein for spermatogenesis.

### Conditional *Ssb1* deletion leads to increased radiation sensitivity *in vivo*

**Figure 6:**
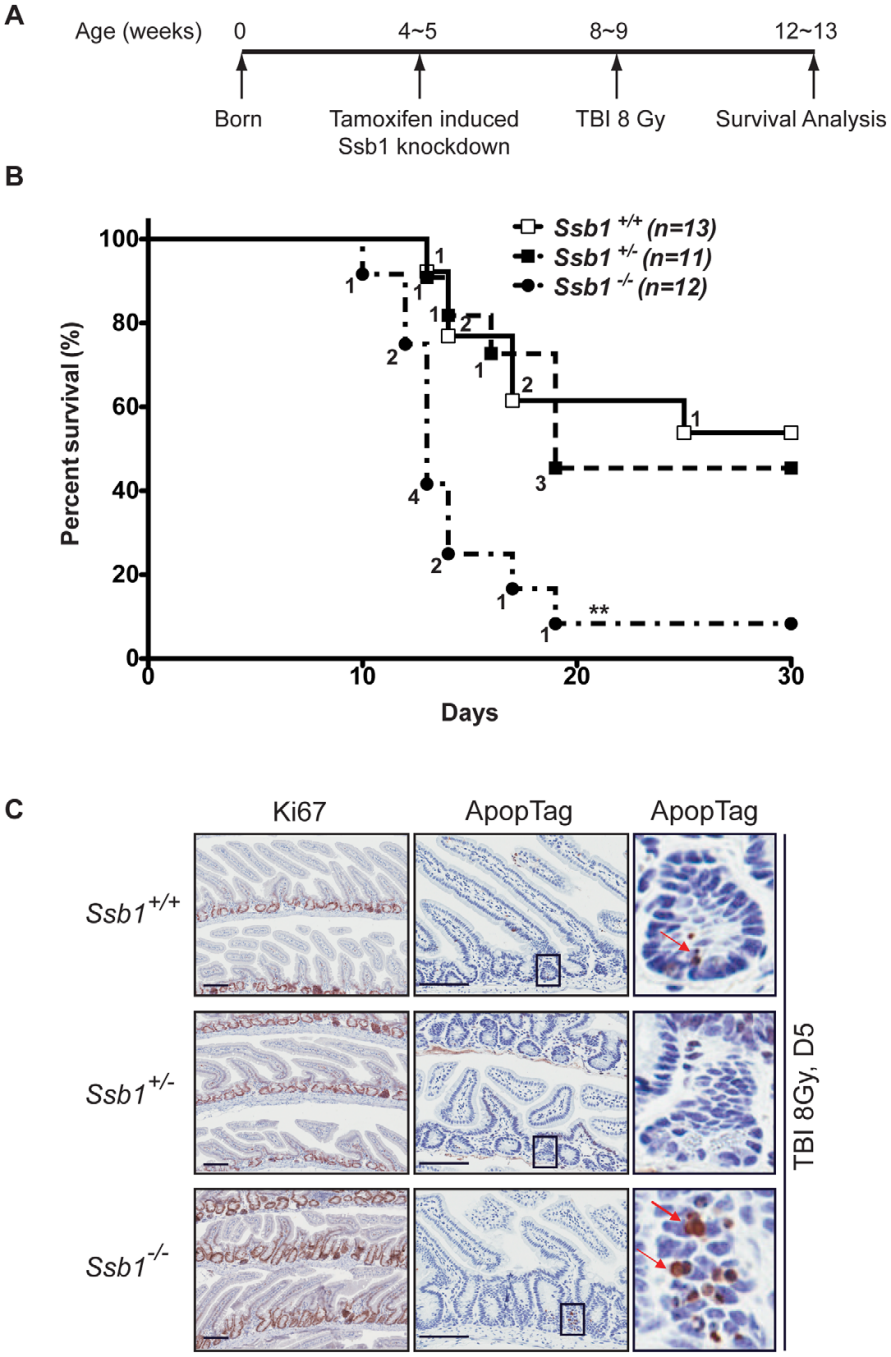
Conditional *Ssb1*-deleted mice are sensitive to IR. (A) Schematic diagram of the radiation challenge assay in *Ssb1* deleted mice. *Rosa26*-CreER^T2^: *Ssb1* ^+/+^, *Rosa26*-CreER^T2^: *Ssb1* ^+/−^ and *Rosa26*-CreER^T2^: *Ssb1* ^−/−^ mice were challenged with 8 Gy of total body irradiation (TBI). The acute lethal response of mice to TBI was evaluated over a 30-day observation period. (B) Kaplan-Meier survival analysis of irradiated mice. Kaplan-Meier survival curves compared by log-rank (Mantel-Cox) analysis showed significant difference between *Rosa26*-CreER^T2^: *Ssb1* ^−/−^ mice and the other two groups (**P<0.01), while no difference was found between *Rosa26*-CreER^T2^: *Ssb1^+/+^* and *Rosa26*-CreER^T2^: *Ssb1^+/−^* groups. (C) Representative images of Haematoxylin and eosin, Ki67 (cell proliferation) and ApopTag (cell death) staining on small intestine sections from mice at Day 5 post 8 Gy of TBI.

To assess if conditional deletion of *Ssb1* in mice causes a DNA damage response defect *in vivo*, we challenged *Rosa26*-CreER^T2^: *Ssb1^+/+^*, *Rosa26*-CreER^T2^: *Ssb1^+/−^* and *Rosa26*-CreER^T2^: *Ssb1* ^−/−^ mice with 8 Gy of total body irradiation (TBI) at 4 weeks post tamoxifen-induction and monitored them for up to 30 days post-IR (Figure 6A). Although we observed comparable progressive weight loss in all 3 groups within the first few days of radiation exposure, death events started to occur in the group of irradiated *Rosa26*-CreER^T2^: *Ssb1^−/−^* mice by the 10th day. By day 19, 92% (11 out of 12) of Rosa26-CreER^T2^: *Ssb1* ^−/−^ mice had died. In contrast, in *Rosa26*-CreER^T2^: *Ssb1^+/+^* and *Rosa26*-CreER^T2^: *Ssb1^+/−^* groups, the first death event occurred on the 13^th^ day and more than 50% of mice survived for at least 30 days after irradiation (Figure 6B). Thus, *in vivo* radiation sensitivity was significantly increased in *Rosa26*-CreER^T2^: *Ssb1^−/−^* mice compared to *Rosa26*-CreER^T2^: *Ssb1^+/+^* or *Rosa26*-CreER^T2^: *Ssb1^+/−^* controls based on Kaplan-Meier survival analysis (**P<0.01) (Figure 6B). As injury of the small intestine or bone marrow are the most common causes of death in irradiated mice, we examined these tissues to establish the cause of death in induced *Rosa26*-CreER^T2^: *Ssb1* ^−/−^ mice. At 24 h and 3 days post TBI, the histology of the small intestine was comparable across induced *Rosa26*-CreER^T2^: *Ssb1* ^+/+^, *Rosa26*-CreER^T2^: *Ssb1* ^+/−^ and *Rosa26*-CreER^T2^: *Ssb1* ^−/−^ mice, as assessed by Haematoxylin and eosin, Ki67 and ApopTag immunohistochemical staining (Figure S10A and data not shown). However, at 5 days post TBI, we observed some pathological abnormalities in *Rosa26*-CreER^T2^: *Ssb1* ^−/−^ mice, including distended crypt lumina lined by attenuated enterocytes and containing desquamated necrotic cellular debris as well as a small increase of cells near deep crypts with apoptotic bodies (Figure 6C).

Further, we performed complete blood count (CBC) analysis on peripheral blood of these mice to assess hematologic radiation toxicity, but no significant difference between the groups was observed (Figure S10B).

To assess whether Ssb1 deficiency affects radiosensitivity in other tissues, we also isolated and exposed thymocytes to various doses of IR (1–6 Gy). We observed increased radiosensitivity in *Ssb1* ^−/−^ thymocytes as assessed by Annexin V and 7-AAD staining (Figure S11). Taken together, these data indicate that depletion of Ssb1 leads to increased radiosensitivity *in vivo* and in thymocytes *in vitro*.

### Increased genomic instability in conditional *Rosa26*-CreER^T2^: *Ssb1* ^−/−^ mice

**Figure 7:**
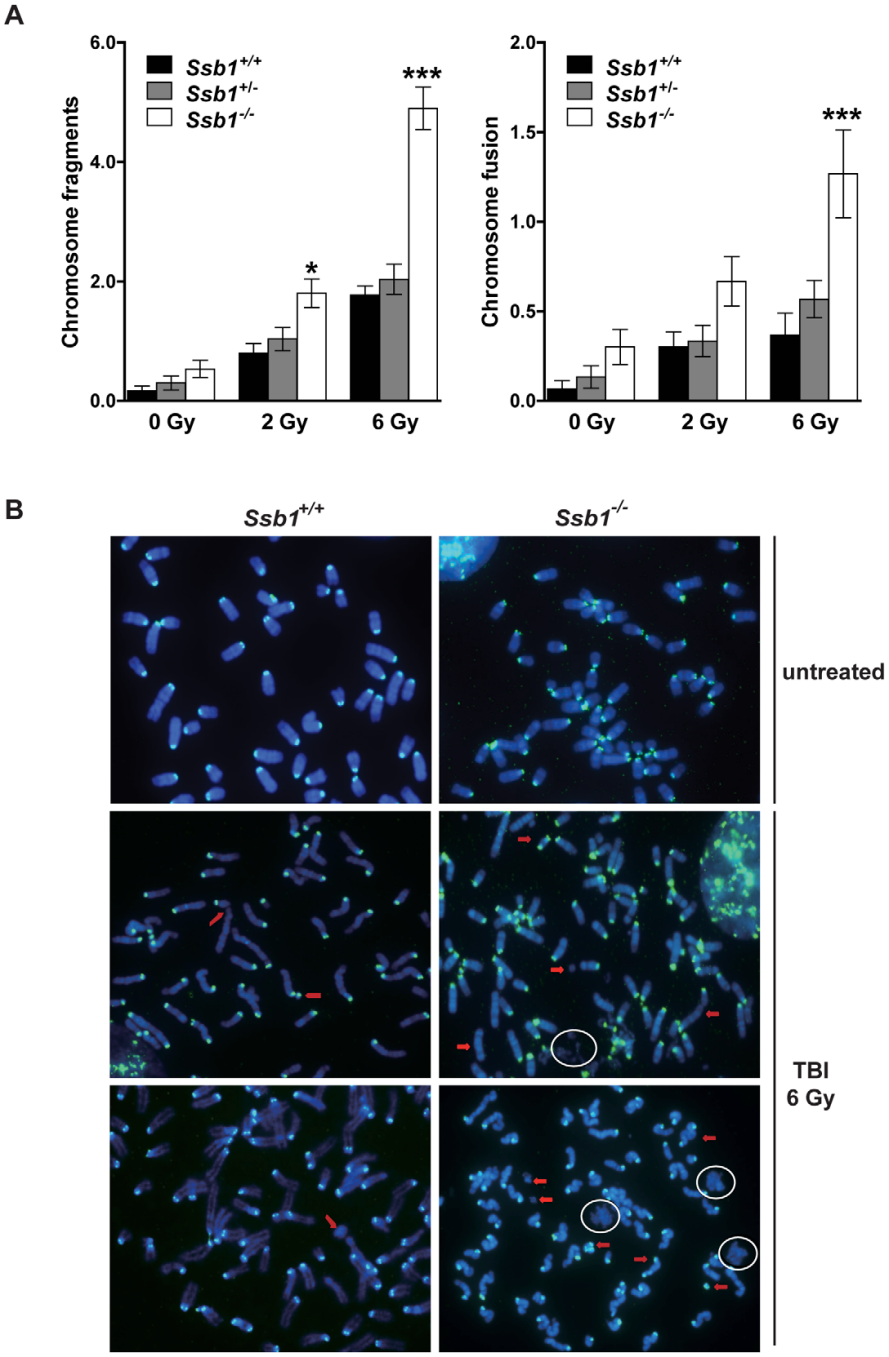
Increased genomic instability in irradiated bone marrow metaphases from total body irradiated mice. Five weeks after tamoxifen induction, nine-week-old *Rosa26*-CreERT2: *Ssb1* ^+/+^, *Rosa26*-CreER^T2^: *Ssb1* ^+/−^ and *Rosa26*-CreER^T2^: *Ssb1* ^−/−^ mice were given 2 or 6 Gy of TBI and kept for 24 h before bone marrow collection. Metaphases were prepared directly from bone marrow cells of demicolcine-treated mice for fluorescence in situ hybridization (FISH) analysis. (A) Quantification of chromosomal breakage (fragmentation and fusion) in bone marrow metaphase spreads from *Rosa26*-CreER^T2^: *Ssb1* ^+/+^, *Rosa26*-CreER^T2^: *Ssb1* ^+/−^ and *Rosa26*-CreER^T2^: *Ssb1* ^−/−^ mice at 24 h after 2 and 6 Gy of TBI (*n* = 3 mice per genotype for each condition). (B) Representative images of bone marrow metaphases from mice with indicted genotypes. Red arrows mark some of the chromosomal breakages. Note the presence of DNA debris marked with circles.

To further investigate the increased radiation sensitivity of conditional *Ssb1* null mice, we cytologically examined bone marrow metaphase spreads from *Rosa26*-CreER^T2^: *Ssb1* ^+/+^, *Rosa26*-CreER^T2^: *Ssb1* ^+/−^ and *Rosa26*-CreER^T2^: *Ssb1* ^−/−^ mice at 24 h after 2 and 6 Gy of TBI to assess chromosomal abnormalities. We observed a significant increase in chromosomal breakage, fragmentation and fusion in Rosa26-CreER^T2^: *Ssb1* ^−/−^ bone marrow metaphases upon irradiation, as assessed by fluorescence in situ hybridization (FISH) analysis (Figure 7). These results provide *in vivo* evidence that Ssb1 functions to maintain genomic stability.

### Broad spontaneous tumour spectrum in conditional *Rosa26*-CreER^T2^: *Ssb1* ^−/−^ mice

**Figure 8:**
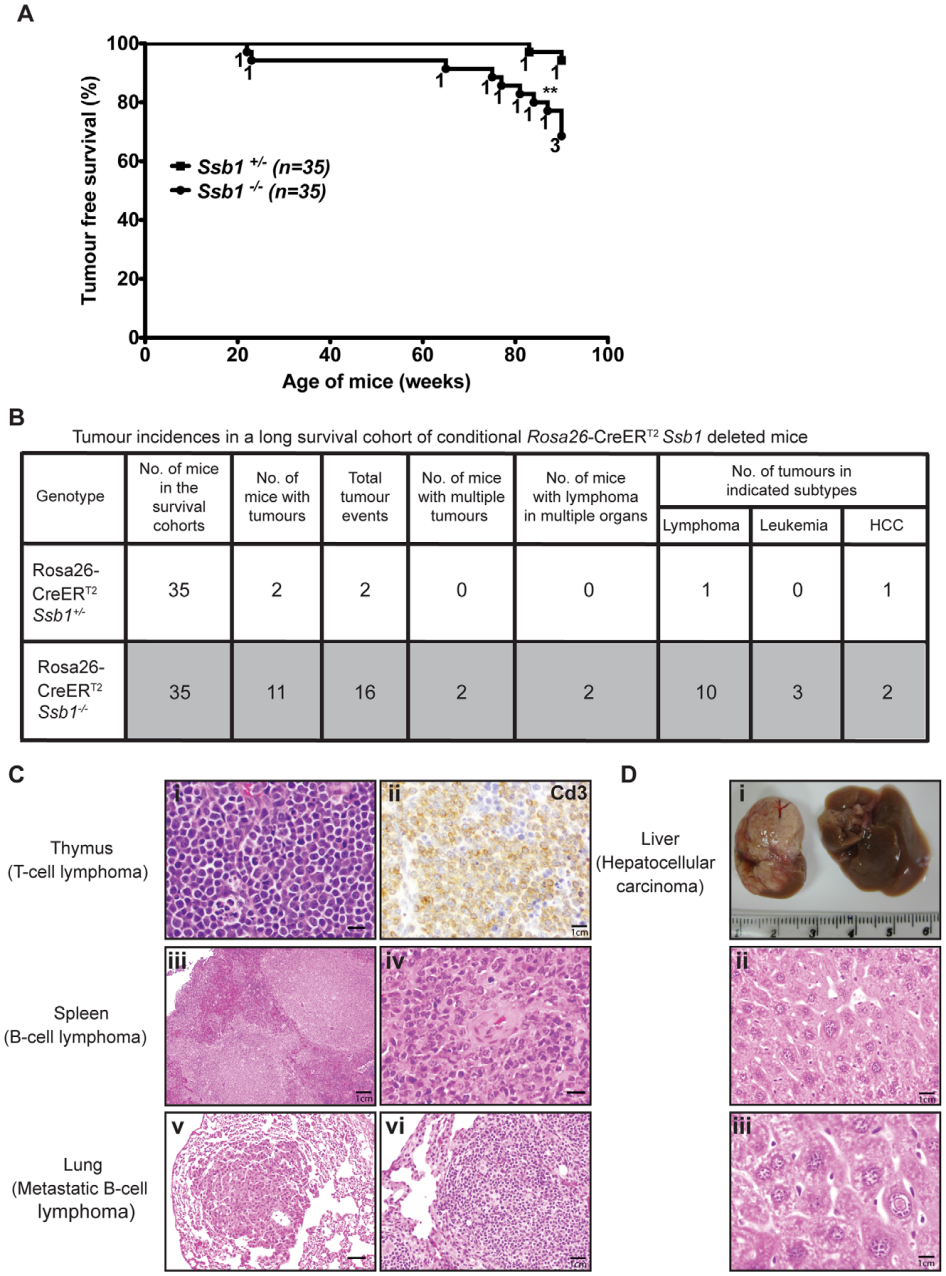
Broad tumour spectrum in conditional *Ssb1*-deleted *Rosa26*-CreER^T2^: *Ssb1* ^−/−^ mice. (A) Long time survival cohort of *Rosa26*-CreER^T2^: *Ssb1* ^+/−^ (*n* = 35) and *Rosa26*-CreER^T2^: *Ssb1* ^−/−^ (*n* = 35) mice were monitored for 86 weeks after *Ssb1* deletion for tumour development. (A) Kaplan-Meier survival analysis showed tumour free survival rate of indicated genotypes over 90 weeks (**P<0.01). (B) Tumour incidence and a spectrum in *Rosa26*-CreER^T2^: *Ssb1* ^−/−^ mice compared with *Rosa26*-CreER^T2^: *Ssb1* ^+/−^ mice. (C) Representative sections have T lymphoma in thymus (top panel) and B lymphomas in spleen (middle panel), which spread to lung in a *Rosa26*-CreER^T2^: *Ssb1* ^−/−^ mice (bottom panel). Representative images of Haematoxylin and eosin stained sections of tumours in indicated organs are shown. Immunohistochemical staining of Cd3 (top panel, right) confirmed T-cell lymphoma in thymus *(Scale bars, i = 40 µm, ii = 120 µm, iii = 20 µm, iv = 40 µm, v = 60 µm, vi = 80 µm)*. (D) Representative image of liver cancer (hepatocellular carcinoma, HCC) in a *Rosa26*-CreER^T2^: *Ssb1* ^−/−^ mouse. Representative images of tumour mass (upper panel) and Haematoxylin and eosin-stained liver section (middle and lower panels) are shown *(Scale bars, ii = 100 µm, iii = 140 µm)*.

To assess whether conditional *Ssb1* deletion would lead to increased cancer susceptibility, we monitored tumour development in age- and gender-matched long-term survival cohorts of *Rosa26*-CreER^T2^: *Ssb1* ^+/−^ (*n* = 35) and *Rosa26*-CreER^T2^: *Ssb1* ^−/−^ (*n* = 35) mice. No significant difference in body weight was found between *Rosa26*-CreER^T2^: *Ssb1^+/−^* and *Rosa26*-CreER^T2^: *Ssb1* ^−/−^ mice over the 86 week observation period post-*Ssb1* deletion (Figure S9). However, during this period, 11 out of 35 (31.4%) *Rosa26*-CreER^T2^: *Ssb1* ^−/−^ mice developed tumours, in contrast to only 2 out of the 35 (5.7%) *Ssb1^+/−^* mice, revealing a statistically significant difference (**P<0.01) in tumour-free Kaplan-Meier survival analysis (Figure 8A). No tumours were observed in a Cre-negative control group (*Ssb1^fl/fl^* mice, *n* = 10) treated with an identical tamoxifen dose or in a vehicle (olive oil: ethanol at 19∶1 ratio) treated *Rosa26*-CreER^T2^: *Ssb1^+/−^* control group (*n* = 5). In the 11 *Rosa26*-CreER^T2^: *Ssb1* ^−/−^ mice that developed tumours, we observed a broad tumour spectrum (Figure 8B) including splenic and metastatic B lymphomas, T cell lymphoma in thymus (Figure 8C), hepatocellular carcinoma, (HCC, Figure 8D) and B or T lymphoblastic leukemia (Figure S12). We also observed p53 stabilization, which is most likely an indication of the presence of mutated p53, in a high proportion of tumours (9 of 11 *Ssb1* ^−/−^ tumours and 2 of 2 *Ssb1^+/−^* tumours) when compared with adjacent normal tissue from the same mice (Figure S13 and Figure S14). Moreover, in the two tumours observed in *Rosa26*-CreER^T2^: *Ssb1* ^+/−^ mice, the Ssb1 protein was undetectable by immunohistochemical staining, indicating possible loss of heterozygosity (LOH) of the other *Ssb1* allele in these tumors (Figure S14). Taken together, these data indicate that Ssb1 prevents tumor formation *in vivo*.

## Discussion

Previous studies using siRNA depletion in human cells have reported a role for SSB1 in the proper co-ordination of DNA repair in response to IR [7], [8], [9], [10]. By disrupting the major protein coding exons of *Ssb1* in mice, including the OB-fold domain, we have created mouse models to study the physiological function of Ssb1 *in vivo*, and describe a wide spectrum of phenotypes upon *Ssb1* deletion during embryogenesis and in adult and aged mice.

Major unexpected findings include novel roles of Ssb1 in the regulation of lung and skeletal development, as constitutive germline ablation of *Ssb1* resulted in immature alveolar differentiation and multiple skeletal defects encompassing the ribs, craniofacial skeleton, and limbs. Interestingly, a handful of other DNA repair factors have been linked to roles in skeletogenesis: patients with Rothmund-Thompson and Rapadilino syndrome, who have mutations in the DNA helicase RECQ4, exhibit some skeletal defects in the limb [25]; patients with mutations in the repair-associated proteins Ctbp-interacting protein (CtIP/RBBP8), Centrosomal Protein 152 (CEP152), microcephalin1 (MCPH1), or Ataxia-Telangiectasia Related (ATR) exhibit dwarfism and a characteristic “bird-shaped” face with micrognathia, which is similar to the craniofacial phenotype we observe in *Ssb1* ^−/−^ mice [26], [27], [28], [29]. Similarly, patients with Nijmegen Breakage Syndrome (NBS), who have mutations in the MRN complex protein NBS-1, also have similar craniofacial abnormalities [30]. Previously, we demonstrated an interaction between SSB1 and NBS1, which, in *in vitro* studies, was abrogated by NBS-1 mutations observed in patients [31]; therefore it is tempting to speculate that SSB1 may be involved in some of the craniofacial phenotypes of this disorder. However, the broad spectrum of skeletal phenotypes in Ssb1-deficient mice is more pronounced than those reported for any of these human syndromes. This, together with the absence of obvious defects in signalling and repair of IR-induced DNA damage in both MEFs and absence of CSR defects in B cell-specific *Ssb1^−/−^* mice, may suggest additional functions of *Ssb1* during embryogenesis that are outside of DNA repair.

Skeletal patterning is a complex process, and involves the spatial and temporal co-ordination of a number of developmental signalling pathways, including Hedgehog (in particular Indian Hedgehog [Ihh] and Sonic Hedgehog [Shh]), Bone Morphogenic Protein (BMP) and the Transforming Growth Factor Beta (TGF-β) family, Fibroblast Growth Factor (FGF) and Wnt signalling [32], [33]. Not surprisingly, a plethora of proteins have been implicated in skeletogenesis. During vertebrate skeletal development, mesenchymal condensations (known as somites) differentiate into the sclerotome and dermomyotome [34], [35]. Whilst the sclerotome differentiates into chondrocytes, which form the ribcage and axial skeleton, the dermomyotome further differentiates into the skin (dermatome) and muscle (myotome). Correct outgrowth and differentiation has been shown to be dependent on signalling from each of these compartments [33], [34], [35].

Interestingly, the rib-cage phenotype we observe in *Ssb1* ^−/−^ skeletons bears striking similarity to that of targeted disruption of the myotome regulator Myf5 [36], [37]. In Myf5-deficient mice, a similar lack of ossification in the ribcage and “floating-rib” phenotype is observed, with a partial or complete lack of ossification of the dorsal region of the ribcage, combined with micrognathia [36], [37]. *Myf5* ^−/−^ mice also die perinatally, but do not show the same degree of hindlimb defects that we observe in *Ssb1* ^−/−^ mice. Intriguingly, *Myf5* is one of the genes hypothesized to have a causal role in cerebro-costo-mandibular syndrome, a rare multiple congenital anomaly syndrome characterized by absent ossification of the posterior rib-cage and micrognathia [38], [39]. Strikingly, cerebro-costo-mandibular syndrome patients also usually exhibit lung hypoplasia, due to improper development of the lung inside a poorly formed rib-cage, and have a poor prognosis for survival [40]. In addition, this disorder has also been associated with hearing defects, variable palate clefting, and sometimes mental retardation [38], [39], [40]. Although limb-patterning defects have not been described for this disorder, given the striking similarity in other phenotypes, *Ssb1* may prove an interesting new candidate gene for this disorder.

Bone development can occur through two major processes, endochondral ossification, where a cartilage precursor template is laid down prior to bone formation, or intramembranous ossification, where mesenchymal cells condense and directly transition to form bone [41], [42]. Whilst endochondral ossification is the process responsible for skeletal formation in the majority of the axial and appendicular skeleton, intramembranous ossification is restricted to parts of the skull, including the cranial vault, and maxillo-mandibular bones [41], [42]. The skeletal outgrowth and patterning defects observed in *Ssb1* ^−/−^ mice suggest that Ssb1 is important for endochondral ossification. During the preparation of this manuscript, another report of the critical role of Ssb1 in skeletogenesis was published, where the authors had used a similar genetic targeting approach to delete *Ssb1* in the mouse [43]. Interestingly, the authors described an almost identical skeletal phenotype to this report, with a similar lack of ossification of the rib-cage, micrognathia, timpanic ring malformation and variably-penetrant oligodactyly. In addition, they also reported clefting of the palate, which we observed in two cases but not in others. However, although both mouse models were generated in C57BL/6 mice, craniofacial phenotypes can be heavily affected by sometimes-subtle strain differences [44]. Intriguingly, the role of Ssb1 in skeletogenesis was attributed to p53-dependent apoptosis at E12.5 throughout the somites and limb, and a partial rescue of these phenotypes was observed upon crossing to a *p53* ^−/−^ background. In the case of combined Ssb1 and p53 ablation, however, although the hindlimb digit patterning and ribcage structure was substantially rescued, a distinctive lack of ossification was still evident, particularly in the dorsal extremities of the ribs abutting the vertebrae [43]. This suggests that the *Ssb1* ^−/−^ phenotype cannot be solely attributed to apoptosis, and that some steps in the later stages of endochondral ossification are dependent on Ssb1. Interestingly, the authors did not observe differences in canonical chondrogenic and osteogenic markers by microarray analysis on E18.5 sternum chondrocytes and calvarial osteoblasts [43]. However, the late time point of analysis and tissue origin of these cell lines may have affected the outcome of this study. Indeed, calvarial osteoblasts form through intramembranous, not endochondral ossification [41], and sternum development was not as severely affected as the rest of the rib-cage in *Ssb1* ^−/−^ embryos. It will therefore be of great interest to more rigorously investigate the role of Ssb1 in bone development, and to determine the precise mechanisms that lead to bone-specific apoptosis observed in these mice.

While the development defects in germline *Ssb1* knockout mice were surprising, effects of inducible ablation of Ssb1 in adult mice revealed phenotypes more relevant to the proposed role of Ssb1 in maintaining genomic stability, as we observed defects in spermatogenesis, increased radiation sensitivity, increased genomic instability as well as an increased tumour incidence in induced *Ssb1* ^−/−^ mice. Spermatogenesis in the mouse commences postnatally at day 7 and by day 35 post-natal mature sperm can be found within the seminiferous tubules. One round of spermatogenesis takes approximately 28 days and it is a continuous process within the testes. The major phases of spermatogenesis are mitosis, meiosis, and post-meiotic germ cell maturation, which last 11, 10 and 14 days, respectively [45]. We commenced induction of *Ssb1* deletion at the age of 28 days, which is at the late stage of meiosis during the first wave of spermatogenesis. We observed a variable degree of testicular degeneration and defective spermatogenesis, which led to smaller sized testes and reduced fertility in conditional *Ssb1^−/−^* adult male mice. The increased number of apoptotic spermatocytes in testes and premature sloughing of germ cells into the epididymis may be the cause of reduced fertility. The observation of a degree of phenotypic variation between conditional *Ssb1* ^−/−^ mice suggests that the severity of the fertility defects was dependent on the degree of testicular degeneration, which may be correlated with the variation in the residual amount of Ssb1 protein between different mice after Cre-recombination. Further investigation of the function of Ssb1 in spermatogenesis is beyond the scope of this first report, but it would be of great interest to study testicular defects in testis-specific *Ssb1*-deleted mice.

Aside from meiotic chromosome rearrangement, physiological programmed DSBs are also generated during Class Switch Recombination (CSR) in mature antigen-stimulated B lymphocytes [17]. CSR involves programmed DNA rearrangements within the Ig heavy chain locus of B-lymphocytes to switch from IgM to other Ig isotypes [17]. Splenic B cells with *Ssb1^−/−^* specific deletion showed similar *ex vivo* induced switching from IgM to IgG1. This lack of a CSR defect in B-cell specific *Ssb1* knockout mice was unexpected, and may be due to functional compensation by Ssb2 as we observed dramatically up-regulated Ssb2 protein levels in bone marrow and spleen from *Rosa26*-CreERT2: *Ssb1* ^−/−^ mice and B cells from *Cd19*-Cre^+^: *Ssb1^−/−^* mice. However, we suspect the potential compensation of Ssb2 might not be sufficient to compensate for all lost Ssb1 functions in the long term or in tissues such as testes, where Ssb2 is already abundantly expressed. To investigate this aspect, a double inducible knockout mouse model of *Ssb1* and *Ssb2* is under investigation in our group, which will provide insight into how the Ssbs are functionally related in DNA repair.

A major role of DDR proteins, particularly crucial HR proteins, in mammalian cells is to maintain genomic integrity [46]. Not surprisingly, the impairment of this process increases cancer risk [19], [20], [21], [22], [47], [48], [49], [50]. Interestingly, the increased radiation sensitivity and chromosomal instability in total body irradiated conditional *Ssb1* ^−/−^ mice demonstrate the importance of Ssb1 in the maintenance of genomic integrity. Further confirming the role of Ssb1 in genomic stability was the increased incidence of spontaneous tumor formation in aged conditional *Ssb1* ^−/−^ mice compared with their heterozygous littermates, revealing a potential tumor suppressor function of Ssb1 *in vivo*. Notably, we were unable to observe a defect in γ-H2ax induction or clearance, nor in Atm signaling in response to ionizing radiation in isolated *Ssb1* ^−/−^ MEFs; however, this does not rule out a potential role of Ssb1 in these processes in a context- or tissue-specific manner.

In conclusion, our results highlight a novel, and non-redundant role of Ssb1 in embryonic development, which may be due to a function independent of its previously described role in DNA repair. Furthermore, our conditional deletion studies of *Ssb1* in adult mice highlight the importance of Ssb1 in maintaining some aspects of genome stability and may represent tissue-specific and context-dependent roles of this protein in the adult mouse.

## Materials and Methods

### Generation of targeting construct

To target the mouse *Ssb1* allele, a targeting construct was engineered with unidirectional lox-P sites flanking exons 3–6 of mouse *Ssb1*, which encompasses the DNA-binding OB-fold domain of the protein. A neomycin resistant cassette (PGK-NEO), necessary for gene targeting in mouse ES cells, was flanked by FRT recombination sites and situated within the lox-P flanked region (Figure S1A). Genomic targeting of the construct was performed in C57BL/6J ES cells using standard homologous recombination and blastocyst manipulation techniques. Gene targeting was confirmed by Southern blot using 5′ and 3′ probes situated outside the targeting vector, in addition to an internal *neo* probe following restriction digest of genomic DNA using *HindIII*, *SacI* or *ScaI* restriction enzymes. Generation of *Ssb1* floxed/neo (flneo) mice was a contracted service performed by Ozgene Pty Ltd (Perth, Australia).

### Generation of constitutive *Ssb1* knockout mice

*Ssb1* floxed (fl) mice were generated by first crossing *Ssb1* targeted mice against FLPe recombinase transgenic mice to remove the neomycin cassette, and subsequently backcrossed onto a C57BL/6J strain to remove the FLP transgene. To generate constitutive germline deletion of *Ssb1, Ssb1^fl/fl^* mice were crossed against CMV-Cre (TgN(CMV-cre)1Cgn) transgenic mice that have been described previously [51]. Offspring containing the *Ssb1* null (−) allele were backcrossed to the C57BL/6J strain to segregate the *Ssb1* null allele and Cre transgene. *Ssb1^+/−^* heterozygous mice were intercrossed to generate *Ssb1^−/−^* animals. *Ssb1* ^+/+^ and *Ssb1* ^+/−^ embryos were indistinguishable at the phenotypic level and were used interchangeably for some experiments as explicitly stated in the text.

### Generation of conditional *Ssb1* knockout mice

To generate conditional and ubiquitous *Ssb1^−/−^* mice, *Ssb1^fl/fl^* mice were crossed against *Rosa26*-CreER^T2^ transgenic mice (Figure S7) [18], [52]. Double transgenic progeny carrying both the *floxed* and *Cre* transgenes (*Rosa26*-CreER^T2^: *Ssb1^fl/+^*) were subsequently crossed to the *Ssb1^fl/fl^* mouse line to generate experimental animals (*Rosa26*-CreER^T2^: *Ssb1^fl/+^* and *Rosa26*-CreER^T2^: *Ssb1^fl/fl^*). Induction of *Ssb1* knockout was performed by intraperitoneal (IP) injection of 1 mg tamoxifen/mouse for 5 consecutive days into 4 week-old experimental animals. Cre-mediated excision was verified in a number of tissues by both genotyping PCR and western-blot (Figure S8).

To determine if Ssb1 plays a role in class switch recombination (CSR), we crossed *Ssb1^fl/fl^* mice with *Cd19*-Cre transgenic mice to conditionally delete *Ssb1* in B cells [53].

### Animal husbandry and ethics

All experimental animals were maintained on a C57BL/6J strain, and were housed at 25°C with a 12 h light/12 h dark cycle. All experiments were performed in accordance with the Queensland Institute of Medical Research animal ethics guidelines.

### Genotype analysis

Genotyping was performed using genomic DNA extracted from tails. The sequences of PCR primers for genotyping *Rosa26*-CreER^T2^ mice are: 5′-TGTGGACAGAGGAGCCATAAC-3′ (forward primer) and 5′-CATCACTCGTTGCATCGACC-3′(reverse primer). As expected, PCR amplification of the 356-bp *Rosa26*-CreER^T2^-specific product reliably identified transgenic mice. Assessment of the *Ssb1* gene before and after Cre recombination was performed by PCR designed to detect if the floxed-sequence had been deleted via Cre/loxP recombination. Two different reverse PCR primers were used, together with a common forward primer, result in 482, 360 and 118-bp PCR products, specific for *Ssb1* floxed, wild-type, and null alleles, respectively (Figure 2A). The sequences of the common forward primer for *Ssb1* wild type, floxed and null allele is: 5′-GCTTTGCTTCTGTTCCTTTACCT-3′. The reverse primer for *Ssb1* wide-type and floxed alleles is 5′-ACAACCTTTGAACACTGAAGC-3′and for the *Ssb1* null allele is 5′-GAAATGGATTCCGAGCTCAA-3′.

### Skeletal preparations

Alcian Blue and Alizarin Red whole-mount skeletal preparations were performed as described previously [54] on E18.5 embryos. Skeletal Preparations were imaged on a Nikon SMZ45 dissecting microscope equipped with a Nikon 5MP colour camera.

### Western blot

For protein extraction, tissue samples were homogenized in RIPA lysis buffer (50 mM Tris-HCl pH 7.4, 150 mM NaCl, 1% NP40, 0.25% Na-deoxycholate, 1 mM PMSF, 1× Roche complete mini protease inhibitor cocktail, 1× Pierce phosphatase inhibitor cocktail). Western blotting was performed as described previously [3] with the following antibodies: sheep anti-SSB1 (1∶1000), rabbit anti-SSB2 (1∶250), mouse anti-β-actin (Sigma, 1∶10,000), mouse anti-phosphorylated-ATM serine1981 (Cell Signaling, 1∶1000) and rabbit anti phosphorylated-p53 serine 15 (Cell Signaling, 1∶1000). Detection of the signals with the chemiluminiscence reagent (Super Signal West Pico, Pierce) was carried out using the LAS-4000 imaging system (Fujifilm Life Science).

### Alveolar lumen and septa quantitation

Images of Haematoxylin and eosin stained lung sections from *Ssb1* control (*Ssb1* ^+/+^, *Ssb1* ^+/−^, *n* = 3) and *Ssb1* ^−/−^ (*n* = 4) were analysed using Image J software (*rsbweb.nih.gov/ij/)* on four representative images for each lung, with care taken not to include areas with conducting airway. Briefly, images were converted to greyscale and thresholded equally across images from control and *Ssb1^−/−^* lungs to highlight alveolar septa. The average area occupied by septa and airspace was calculated and subjected to statistical analysis.

### Quantitative real-time PCR on lungs

The right lobes of *Ssb1* ^+/+^, *Ssb1* ^+/−^ and *Ssb1* ^−/−^ lungs were homogenized and RNA extracted using the RNeasy mini kit (Qiagen), followed by *DNAse I* (New England Biolabs) digestion to remove genomic DNA contamination. 2 µg of RNA was used for first-strand cDNA synthesis using random primers (Life Technologies) and Superscript III reverse transcriptase (Life Technologies). qRT-PCR was performed using Light Cycler 480 Sybr green I mastermix (Roche Applied Science) on a Light Cycler 480 Real-time PCR system (Roche Applied Science). Primer sequences for *Cc10*, *Foxj1*, *Cd31*, *Pdpn*, *Sftpa, Sftpb*, *Sftpc* and *Sftpd* have been described previously [55], [56]. *Aqp5* and *β-Actin* primer sequences were as follows: *Aqp5*, 5′-CTGCGGTGGTCATGAATC-3′ (forward) and 5′-CTACCCAGAAGACCCAGTGA-3′ (reverse); *β-Actin*, 5′-GGCTGTATTCCCCTCCATCG-3′ (forward) and 5′-CCAGTTGGTAACAATGCCATGT-3′ (reverse). Negative controls with no template and no reverse transcriptase were also included and used in qRT-PCR reactions to ensure no contaminating genomic DNA was present.

### Mouse embryonic fibroblast isolation and 3T3 growth assays

Mouse embryonic fibroblasts (MEFs) were isolated from E13.5 embryos from *Ssb1* ^+/−^ intercrosses as described previously [57]. At least three independent embryos per condition were used for analysis. For 3T3 fibroblast growth assays, *Ssb1* ^+/+^ and *Ssb1* ^−/−^ cell lines were seeded at passage 2 at a concentration of 0.5×10^6^ cells/10 cm dish. Cells were trypsinised, counted and re-seeded every 3 days at the same concentration to monitor relative changes in growth at each passage.

### Immunofluorescence and microscopy

Cells were plated on glass coverslips and used at approximately 70 percent confluency. Immunofluorescence with the γ-H2AX antibody (Millipore) was performed as described previously [7]. For γ-H2ax foci quantitation, 50 cells for each MEF cell line (*n* = 2 *Ssb1* ^+/+^, 3 *Ssb1* ^−/−^) were scored for those containing >10 foci/cell at the indicated timepoints following 2 Gy of gamma-irradiation, across two independent experiments.

### Gonado-somatic index analysis

The testes from *Rosa26*-CreER^T2^: *Ssb1* mice were dissected out and weighed with an analytical balance (Mettler AT261). The gonado-somatic index was determined according to the formula: Gonado-Somatic Index (GSI) = (Gonad weight/total body weight) X 100, where gonad weight = (weight of the right testis+ weight of the left testis)/2 [23].

### Class switch recombination (CSR) analysis

Splenic B cells were stimulated for IgH CSR to IgG1 using anti-CD40 antibodies plus IL-4 and analyzed by flow cytometry as described previously [58].

### Total body irradiation

Total body irradiation (TBI) was performed using a ^137^Cs source at 108 cGy/min. Mice were placed in plexiglass cages and irradiated in groups of five simultaneously with the indicated doses.

### Florescence *in situ* hybridization (FISH) analysis of chromosomal aberrations

Metaphases were prepared directly from bone marrow cells in demicolcine-treated mice for FISH analysis. Five weeks after tamoxifen induction, nine-week-old *Rosa26*-CreERT2: *Ssb1* ^+/+^, *Rosa26*-CreER^T2^: *Ssb1* ^+/−^ and *Rosa26*-CreER^T2^: *Ssb1* ^−/−^ mice were given 2 or 6 Gy of TBI and kept for 24 h before bone marrow collection. Demicolcine (Sigma, 250 µl of a 200 µg/ml solution) was administered by intraperitoneal injection into each mouse 1 h prior to bone marrow collection. Bone marrow was flushed from each femur and tibia with pre-warmed potassium chloride solution (0.06 M). Fluorescence *in situ* hybridization (FISH) analysis was performed on metaphases using a biotinylated centromere-specific minor satellite probe. Three mice were analyzed for each genotype per condition and thirty metaphases were analyzed per case for chromosome breaks. Within each spread, the number of chromosomal fragments and fusions (identified by the presence of more than one centromere signals) was determined.

### Lymphocyte surface markers staining

Lymphocyte surface makers were measured in peripheral blood samples by flow-cytometric analysis. Following lysis with 0.145 M ammonium chloride to remove red blood cells, the remaining lymphocytes were washed and incubated with APC conjugated anti-Cd3, PerCP-conjugated anti-Cd8, FITC-conjugated anti-Cd4, and PE-conjugated anti-Cd19 (BD Pharmingen), at 4°C for 30 minutes. Cells were washed, resuspended in PBS, and acquired on a FACS Canto II. Data were analyzed with Flowjo software (Ashland, OR, USA).

### Histopathological analysis and immunohistochemistry

Tissues were collected and fixed in 10% buffered formalin fixative or 4% Paraformaldehyde, embedded in paraffin blocks, and 5-µm-thick sections were stained with Haematoxylin and eosin for histological examination. Slides were coded and examined in a blinded fashion by an independent veterinary pathologist. Immunohistochemistry staining was performed following standard procedures. Apoptosis was assessed using the ApopTag peroxidase in situ apoptosis detection kit (Chemicon International), according to the manufacturer's instructions. Stained slides were scanned on Aperio ScanScope XT Slide Scanner and the images were analyzed with Image Scope software.

### Statistical analysis

Data were analyzed with GraphPad Prism software. The student's *t*-test was used for the statistical analysis of embryo weight and length, long bone comparison, qPCR, lung airspace analysis, testis weight, GSI, litter interval, litter size, chromosome breaks and blood cell counting data. Survival curves were plotted using Kaplan-Meier estimates and compared by log-rank (Mantel-Cox) analysis. P values less than 0.05 were considered statistically significant.

## Supporting Information

Figure S1 Generation of *Ssb1* gene-targeted mice. (A) Schematic diagram showing the *Ssb1* gene structure and targeting strategy including the *Ssb1 wild-type* (wt), *Ssb1 targeted* (flneo), *Ssb1 floxed* (fl) and *Ssb1 deleted* (null) alleles. (B) Southern blot confirming correct genomic targeting of *Ssb1 flneo* mice following *ScaI* restriction digest. Samples were probed with both an endogenous probe (enP; top) and neomycin probe (neoP; bottom). Neo cont. designates an unrelated neomycin transgenic mouse used as a positive control. (C) PCR genotyping showing *Ssb1 wild type* (primer 1; P1 and primer 2; P2 in *Ssb1^+/+^*), *Ssb1 flneo* (P1 and P3 in *Ssb1^flneo/+^*), *Ssb1 floxed* (P1 and P2 in *Ssb1^fl/+^*) and *Ssb1 null* (P1 and P4 in *Ssb1^+/−^*) alleles.
(TIF)

Figure S2 Perinatal lethality, growth retardation, micrognathia and cleft palate in *Ssb1^−/−^* embryos. (A) *Ssb1^−/−^* P0 embryos exhibit severe respiratory distress and die within 30 minutes of birth. Note the purple colour of *Ssb1^−/−^* embryos indicating cyanosis. (B) Comparison of crown-rump length of E14.5 and E18.5 *Ssb1^+/+^*, *Ssb1^+/−^* and *Ssb1^−/−^* embryos (*n* = minimum 3 embryos for E14.5; minimum 6 embryos for E18.5 per genotype) (*P<0.05, **P<0.01 ***P<0.001, student's *t*-test). (C) Representative sagittal sections through the heads of *Ssb1^+/+^* (i–iii) and *Ssb1^−/−^* (iv–vi) P0 embryos show a misshapen snout and recessed mandible in *Ssb1^−/−^* embryos. (D) Ventral skull view of E18.5 skeletal preparations with removed mandible showing clefting of the secondary palate in an *Ssb1^−/−^* embryo. *Scale bar = 1 mm*. (E) Magnified view of (D). Note the properly fused palatine processes (arrowhead) in *Ssb1^+/−^* control embryo (left) and lack of palatine process formation in the *Ssb1^−/−^* embryo (right), exposing the underlying presphenoid bone (arrowhead). *Scale bar = 1 mm*. (F) Ventral view of P0 *Ssb1* ^+/−^ and *Ssb1* ^−/−^ heads with removed mandible showing variably penetrant cleft palate between *Ssb1* ^−/−^ littermates. *Scale bar = 1 mm*.
(TIF)

Figure S3 Morphology of *Ssb1* control and *Ssb1^−/−^* embryos. Haematoxylin and eosin staining of sagittal sections of E18.5 embryos showing gross organ morphology. *Scale bar = 2 mm*.
(TIF)

Figure S4 Apoptosis and proliferation in E14.5 and E18.5 *Ssb1^−/−^* lungs. (A) Immunohistological staining of Ki67 in E14.5 (top) and E18.5 (bottom) control (*Ssb1^+/+^, Ssb1^+/−^*) and *Ssb1^−/−^* lungs to mark proliferating cells. (B) ApopTag TUNEL immunohistological staining to mark apoptotic cells in *Ssb1* control and *Ssb1^−/−^* E14.5 (top) and E18.5 (bottom) lungs. *Scale bar = 50 µm*.
(TIF)

Figure S5 Proximal Lung Differentiation in E18.5 *Ssb1^−/−^* lungs. (A) Quantitation of qRT-PCR for proximal differentiation markers *Cc10* (clara cells), *Foxj1* (ciliated epithelial cells) and *Cd31* (endothelial cells). (B) Immunohistological staining for smooth muscle actin (SMA) in *Ssb1* control (*Ssb1^+/+^*, *Ssb1^+/−^*) and *Ssb1^−/−^* E18.5 lungs. *Scale bar = 200 µm*.
(TIF)

Figure S6 Ssb1 is not required for the response to DNA double-strand breaks in mouse embryonic fibroblasts. (A) Cell cycle profiles of *Ssb1^+/+^* and *Ssb1^−/−^* passage 3 MEFs by propidium iodide staining. (B) 3T3 proliferation assay showing growth curves for *Ssb1^+/+^* and *Ssb1^−/−^* MEFs (*n* = 3). Data represent mean ± SEM. (C) Western blot showing Atm signalling activation in *Ssb1^+/−^* and *Ssb1^−/−^* MEFs following 6 Gy of ionizing radiation with indicated antibodies. (D) Immunofluorescence imaging and (E) quantitation for γ-H2ax foci after 2 Gy of ionizing radiation at the indicated timepoints. Data represent mean ± SEM.
(TIF)

Figure S7 Schematic diagram of the conditional *Ssb1* gene targeting strategy. *Ssb1^fl/fl^* mice were bred with *Rosa26-*CreER^T2^ transgenic mice to enable conditional *Ssb1* deletion. *Ssb1* gene deletion was induced by intraperitoneal injection (I.P.) of 1 mg/mouse tamoxifen daily for 5 consecutive days into 4-week-old *Rosa26-*CreER^T2^ *: Ssb1^fl/fl^ mice*.
(TIF)

Figure S8 Conditional Cre recombination mediated *Ssb1* deletion and Ssb2 upregulation in *Rosa26-*CreER^T2^ *: Ssb1^−/−^* mice. (A) PCR genotyping after tamoxifen induced Cre recombination mediated *Ssb1* gene deletion. PCR analysis of recombination of the floxed *Ssb1* allele in heterozygous *Ssb1-floxed-Rosa26-*CreER^T2^ (*Rosa26-*CreER^T2^ *: Ssb1^fl/+^*) mice and homozygous *Ssb1-floxed-Rosa26-*CreER^T2^(*Rosa26-*CreER^T2^ *: Ssb1^fl/fl^*) mice was performed ten days after the final tamoxifen injection. The efficacy of gene interruption in indicated tissues is shown. The PCR products of floxed (fl), wild type (wt) and deletion (null) alleles of *Ssb1* were detected as 482, 360 and 118-bp bands, respectively. (B) Western blot analysis of Ssb1 protein in tissue extracts from mice following Cre recombination. Ssb1 protein levels were analyzed in the indicated tissues ten days after the final tamoxifen injection by immunoblotting with an antibody specific for Ssb1 and ß-actin as a loading control. (C) Western blot analysis of Ssb1 and Ssb2 protein in indicated tissues prepared from *Rosa26-*CreER^T2^ *: Ssb1^+/−^* and *Rosa26-*CreER^T2^ *: Ssb1^−/−^* mice ten days after Cre recombination. *Ssb1^+/−^* and *Ssb1^−/−^* mice were subjected to 6 Gy of total body irradiation (TBI). Indicated tissues were extracted 6 h post irradiation, and Ssb1 and Ssb2 protein levels were analyzed by immunoblotting. Immunoblotting of ß-Actin was used as a loading control.
(TIF)

Figure S9 Comparison of body weights of *Rosa26-*CreER^T2^ *: Ssb1^+/−^* and *Rosa26-*CreER^T2^ *Ssb1^−/−^* mice. (A) Gender distribution comparison of *Rosa26-*CreER^T2^ *: Ssb1^+/−^*and *Rosa26-* CreER^T2^ *: Ssb1^−/−^* mouse cohorts. (B) Comparison of age of tamoxifen induction between cohorts. (C) Comparison of body weights of *Rosa26-*CreER^T2^ *: Ssb1^+/−^* and *Rosa26-*CreER^T2^ *Ssb1^−/−^* mice after tamoxifen injection (*n* = 35).
(TIF)

Figure S10 Histological analysis and complete blood count of mice at 24 h post total body irradiation (TBI). (A) Representative images of Haematoxylin and eosin, Ki67 (cell proliferation) and ApopTag (cell death) staining on small intestine sections from mice at 24 h post 8 Gy of TBI. (B) Complete blood count (CBC) analysis on peripheral blood from mice at 24 h post 8 Gy of TBI. Whole blood samples were processed for counts using Beckman Coulter ACT whole blood counter. Numbers of white blood cell (WBC), red blood cell (RBC), hemoglobin (Hgb), and platelets (Plt) were assessed. *Scale = 100 µm*.
(TIF)

Figure S11 Assessment of radiosensitivity of thymocytes from *Rosa26-*CreER^T2^ *: Ssb1^+/−^* and *Rosa26-*CreER^T2^ *: Ssb1^−/−^* mice. Thymocytes were isolated from mice with indicated genotype and exposed to 1, 3 and 6 Gy of irradiation. (A) Percentage of cell death (Annexin V+/7-AAD+) of *Ssb1^+/−^* and *Ssb1^−/−^* thymocytes at indicted doses of irradiation (*n* = 3, **P<0.01, ***P<0.001; student's *t-*test). (B) Percentage of apoptotic cells (Annexin V+/7-AAD-) at indicated conditions (*n* = 3, **P<0.01, ***P<0.001; student's *t-*test).
(TIF)

Figure S12 B-cell leukemia identified in a *Rosa26*-CreER^T2^ : *Ssb1^−/−^* mouse. (A) Representative flow cytometric analysis on lymphoblasts from peripheral blood (PB). Lymphoblasts were stained as Cd19 (B cell) positive lymphomas. (B) Wright's stain on PB smears showing lymphoblast cluster from a *Rosa26*-CreER^T2^ : *Ssb1^−/−^* mouse (ii) compared with a healthy control littermate (i). Leukocytes featured as large-sized undifferentiated haematopoietic cells with a small basophilic cytoplasm and visible nucleoli *(Scale = 20 µm)*. (C) Lymphocytic leukemia involving the liver. Representative images of Haematoxylin and eosin stained sections (upper panel) and immunohistochemical staining of B220 or Cd3 (lower panel) showing periportal infiltration by B-lymphocytes *(Scale = 50 µm)*. (D) Effacement of the lymph node architecture. Low-power (upper panel) and enlarged views (lower panel) show a periportal lymphocytic infiltrate in the lymph node *(Scale = 50 µm)*.
(TIF)

Figure S13 Representative images of p53 immunohistochemistry staining on tumour sections from *Rosa26*-CreER^T2^ : *Ssb1^−/−^* mice. Tumours developed in indicated organs from *Rosa26*-CreER^T2^: *Ssb1* ^−/−^ mice were stained with p53 antibody (bottom panel) and compared with adjacent normal tissue from the same mice (top panel), *Scale = 100 µm*.
(TIF)

Figure S14 Representative images of Ssb1 and p53 immunohistochemistry staining on tumour sections from *Rosa26*-CreER^T2^ : *Ssb^+/−^* mice. Two tumours observed in *Rosa26*-CreER^T2^: *Ssb1* ^+/−^ mice were stained with Ssb1 and p53 antibodies. Left panel is the control staining of Ssb1 on the respective organs from littermate control of *Rosa26*-CreER^T2^: *Ssb1* ^+/−^ mice. Middle panel is Ssb1 staining from the *Rosa26*-CreER^T2^: *Ssb1* ^+/−^ mice which developed tumours. Right panel is p53 staining of the tumour sections. *Scale = 100 µm*.
(TIF)
