# Supplementary figures and images for: Essential Developmental, Genomic Stability, and Tumour Suppressor Functions of the Mouse Orthologue of hSSB1/NABP2
Source: PLoS Genet. 2013 Feb 7;9(2):e1003298. doi: 10.1371/journal.pgen.1003298 (PMC3567186; doi:10.1371/journal.pgen.1003298)

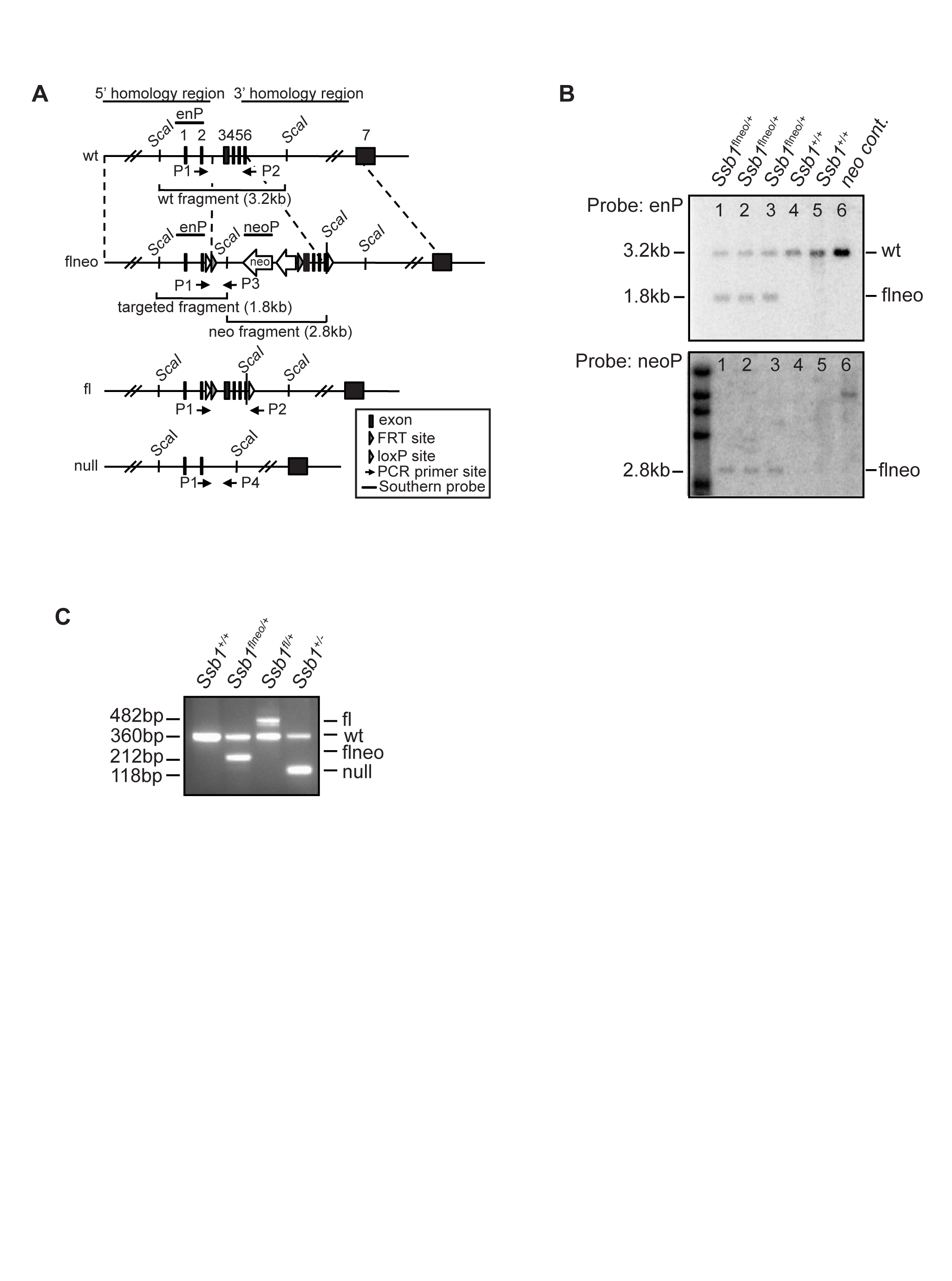

Supplement: Figure S1 — Generation of Ssb1 gene-targeted mice. (A) Schematic diagram showing the Ssb1 gene structure and targeting strategy including the Ssb1 wild-type (wt), Ssb1 targeted (flneo), Ssb1 floxed (fl) and Ssb1 deleted (null) alleles. (B) Southern blot confirming correct genomic targeting of Ssb1 flneo mice following ScaI restriction digest. Samples were probed with both an endogenous probe (enP; top) and neomycin probe (neoP; bottom). Neo cont. designates an unrelated neomycin transgenic mouse used as a positive control. (C) PCR genotyping showing Ssb1 wild type (primer 1; P1 and primer 2; P2 in Ssb1+/+), Ssb1 flneo (P1 and P3 in Ssb1flneo/+), Ssb1 floxed (P1 and P2 in Ssb1fl/+) and Ssb1 null (P1 and P4 in Ssb1+/−) alleles. (TIF) [file pgen.1003298.s001.tif]

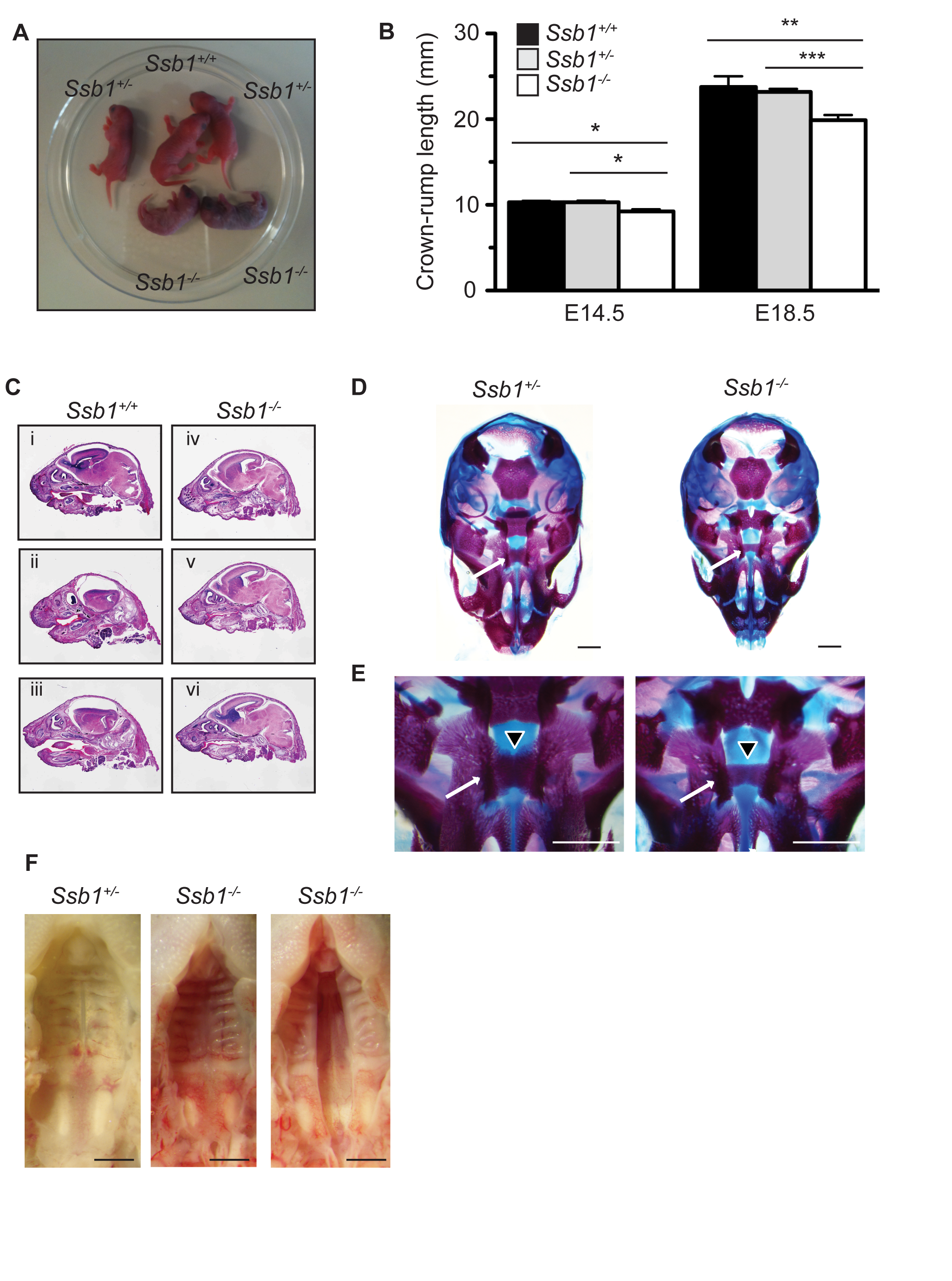

Supplement: Figure S2 — Perinatal lethality, growth retardation, micrognathia and cleft palate in Ssb1−/− embryos. (A) Ssb1−/− P0 embryos exhibit severe respiratory distress and die within 30 minutes of birth. Note the purple colour of Ssb1−/− embryos indicating cyanosis. (B) Comparison of crown-rump length of E14.5 and E18.5 Ssb1+/+, Ssb1+/− and Ssb1−/− embryos (n = minimum 3 embryos for E14.5; minimum 6 embryos for E18.5 per genotype) (*P<0.05, **P<0.01 ***P<0.001, student's t-test). (C) Representative sagittal sections through the heads of Ssb1+/+ (i–iii) and Ssb1−/− (iv–vi) P0 embryos show a misshapen snout and recessed mandible in Ssb1−/− embryos. (D) Ventral skull view of E18.5 skeletal preparations with removed mandible showing clefting of the secondary palate in an Ssb1−/− embryo. Scale bar = 1 mm. (E) Magnified view of (D). Note the properly fused palatine processes (arrowhead) in Ssb1+/− control embryo (left) and lack of palatine process formation in the Ssb1−/− embryo (right), exposing the underlying presphenoid bone (arrowhead). Scale bar = 1 mm. (F) Ventral view of P0 Ssb1 +/− and Ssb1 −/− heads with removed mandible showing variably penetrant cleft palate between Ssb1 −/− littermates. Scale bar = 1 mm. (TIF) [file pgen.1003298.s002.tif]

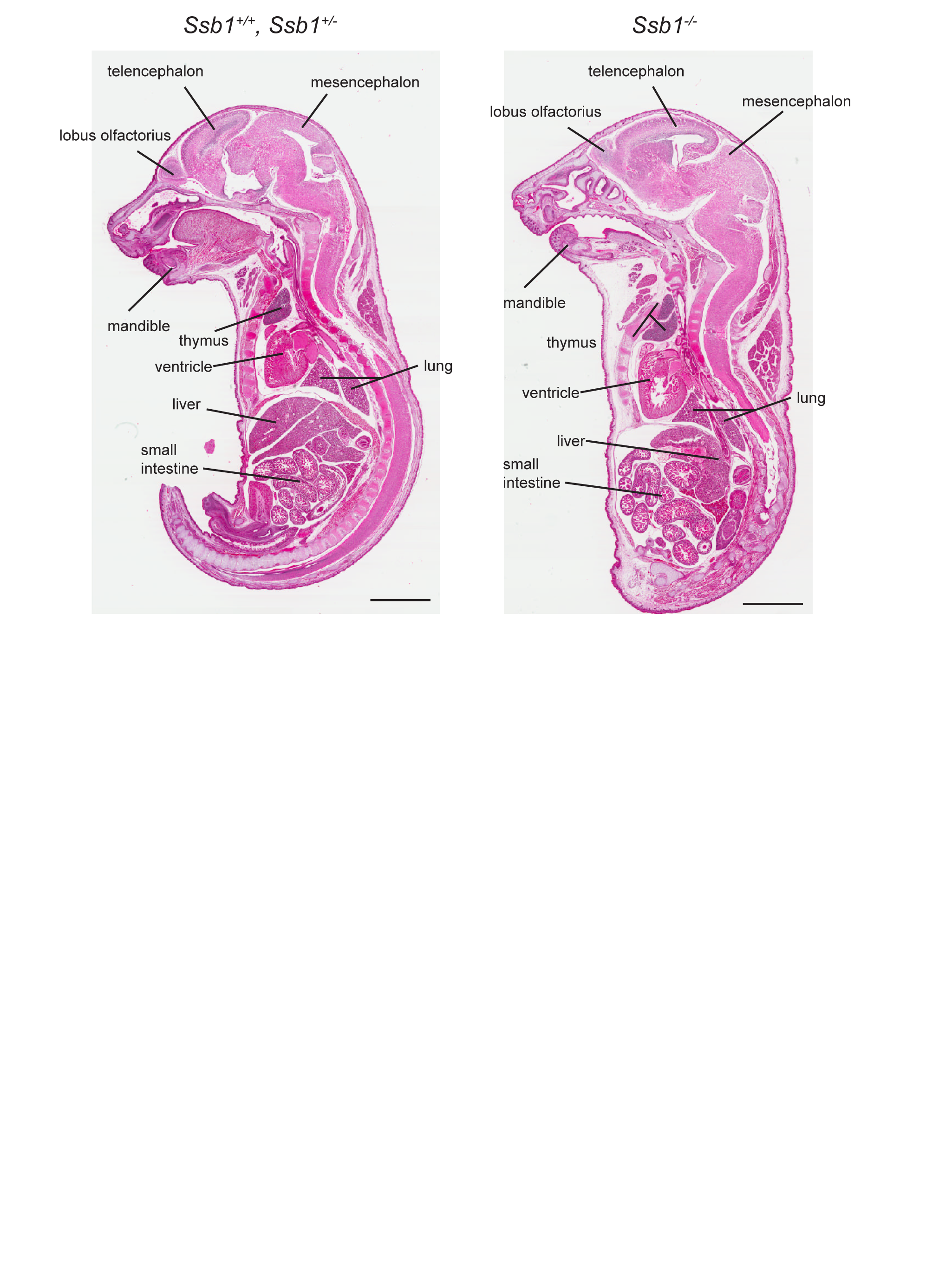

Supplement: Figure S3 — Morphology of Ssb1 control and Ssb1−/− embryos. Haematoxylin and eosin staining of sagittal sections of E18.5 embryos showing gross organ morphology. Scale bar = 2 mm. (TIF) [file pgen.1003298.s003.tif]

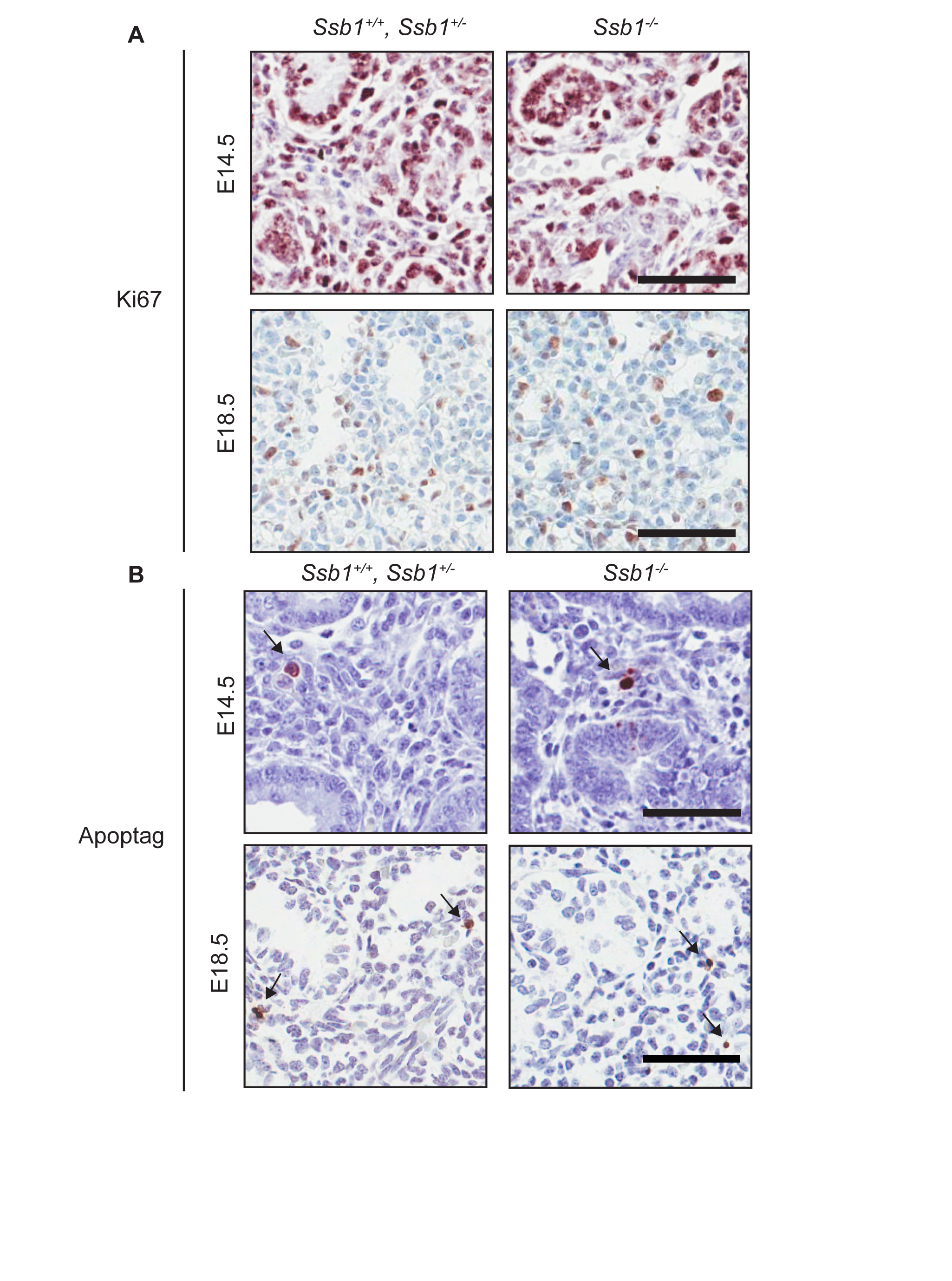

Supplement: Figure S4 — Apoptosis and proliferation in E14.5 and E18.5 Ssb1−/− lungs. (A) Immunohistological staining of Ki67 in E14.5 (top) and E18.5 (bottom) control (Ssb1+/+, Ssb1+/−) and Ssb1−/− lungs to mark proliferating cells. (B) ApopTag TUNEL immunohistological staining to mark apoptotic cells in Ssb1 control and Ssb1−/− E14.5 (top) and E18.5 (bottom) lungs. Scale bar = 50 µm. (TIF) [file pgen.1003298.s004.tif]

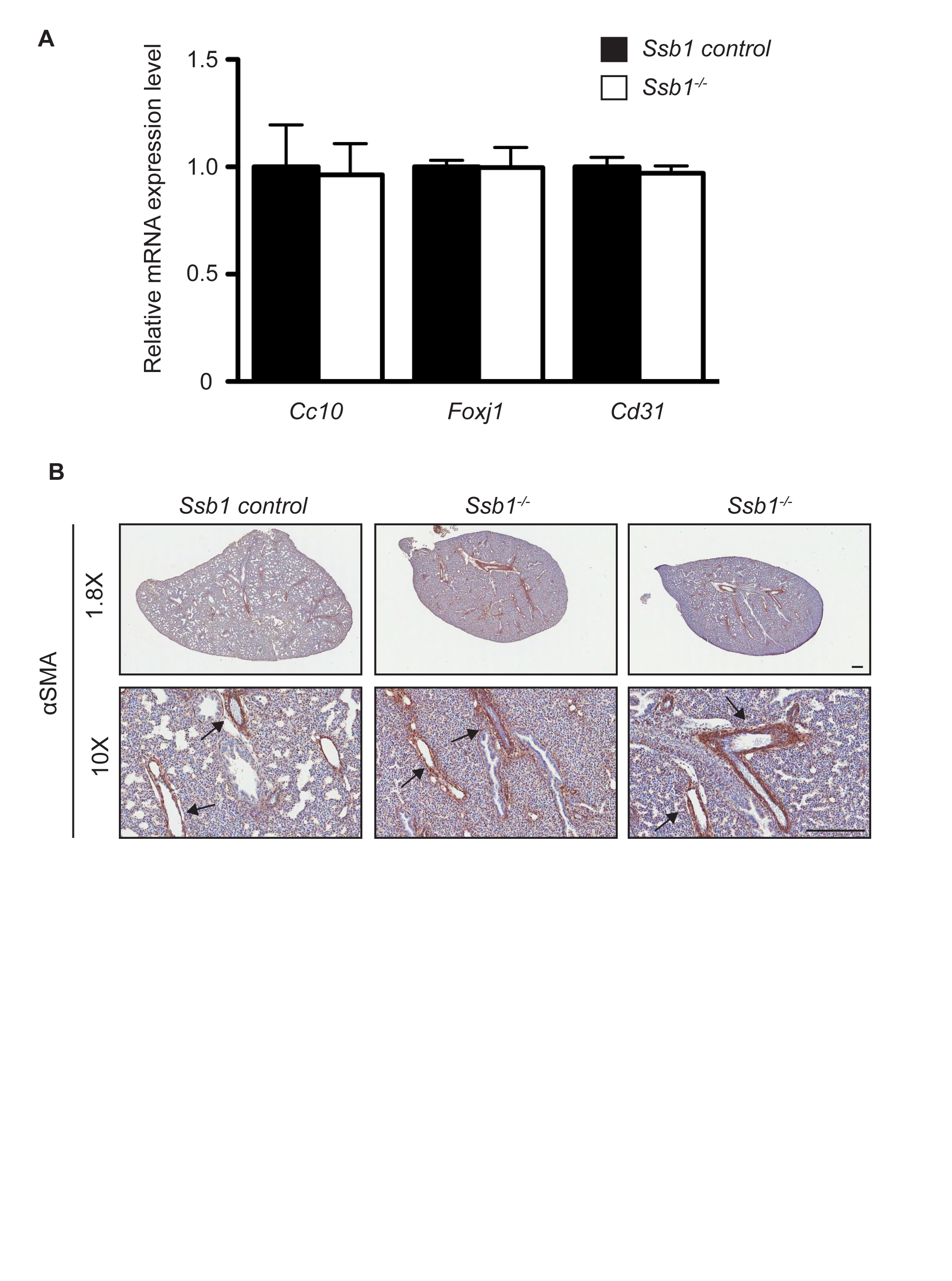

Supplement: Figure S5 — Proximal Lung Differentiation in E18.5 Ssb1−/− lungs. (A) Quantitation of qRT-PCR for proximal differentiation markers Cc10 (clara cells), Foxj1 (ciliated epithelial cells) and Cd31 (endothelial cells). (B) Immunohistological staining for smooth muscle actin (SMA) in Ssb1 control (Ssb1+/+, Ssb1+/−) and Ssb1−/− E18.5 lungs. Scale bar = 200 µm. (TIF) [file pgen.1003298.s005.tif]

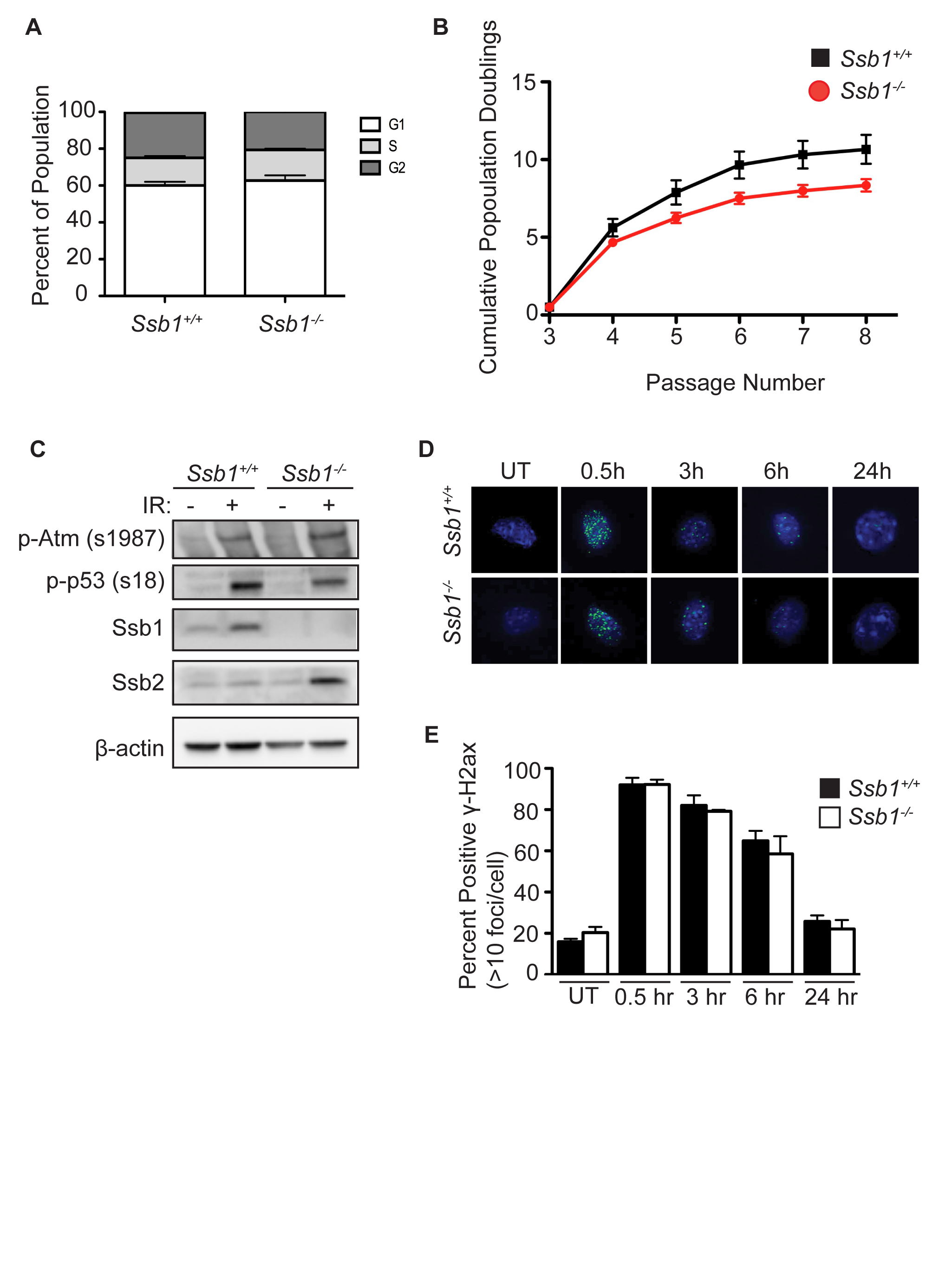

Supplement: Figure S6 — Ssb1 is not required for the response to DNA double-strand breaks in mouse embryonic fibroblasts. (A) Cell cycle profiles of Ssb1+/+ and Ssb1−/− passage 3 MEFs by propidium iodide staining. (B) 3T3 proliferation assay showing growth curves for Ssb1+/+ and Ssb1−/− MEFs (n = 3). Data represent mean ± SEM. (C) Western blot showing Atm signalling activation in Ssb1+/− and Ssb1−/− MEFs following 6 Gy of ionizing radiation with indicated antibodies. (D) Immunofluorescence imaging and (E) quantitation for γ-H2ax foci after 2 Gy of ionizing radiation at the indicated timepoints. Data represent mean ± SEM. (TIF) [file pgen.1003298.s006.tif]

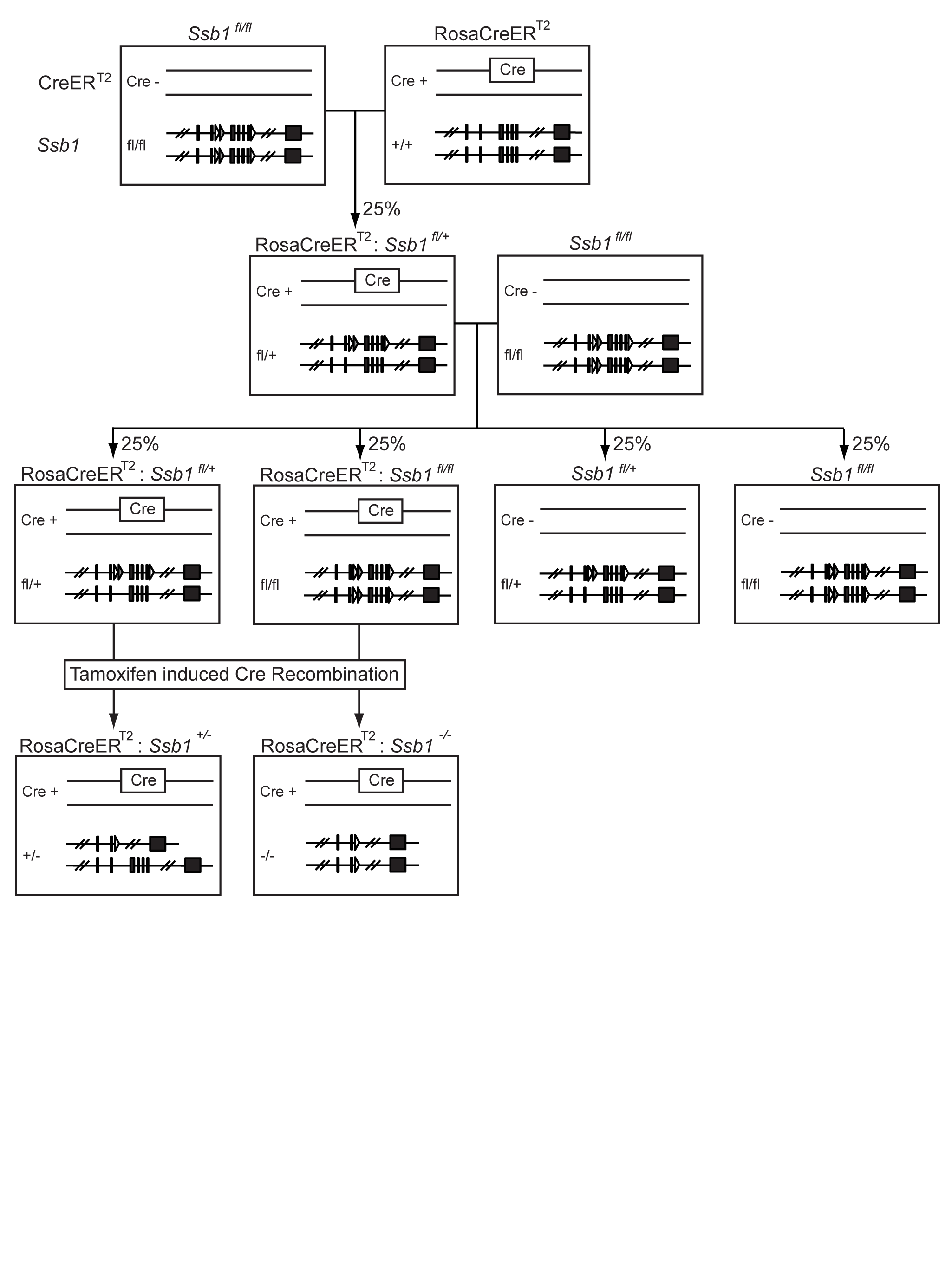

Supplement: Figure S7 — Schematic diagram of the conditional Ssb1 gene targeting strategy. Ssb1fl/fl mice were bred with Rosa26-CreERT2 transgenic mice to enable conditional Ssb1 deletion. Ssb1 gene deletion was induced by intraperitoneal injection (I.P.) of 1 mg/mouse tamoxifen daily for 5 consecutive days into 4-week-old Rosa26-CreERT2 : Ssb1fl/fl mice. (TIF) [file pgen.1003298.s007.tif]

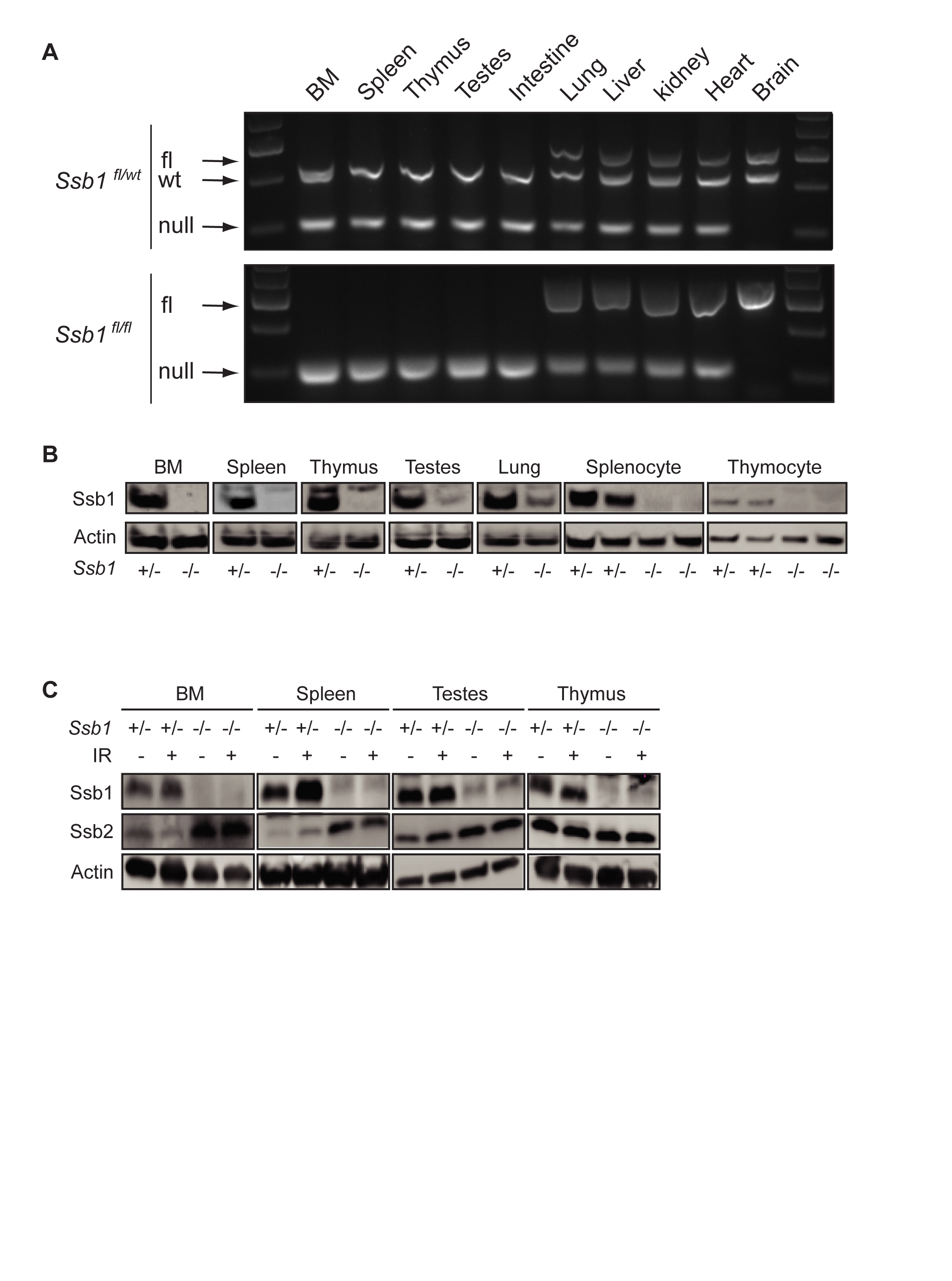

Supplement: Figure S8 — Conditional Cre recombination mediated Ssb1 deletion and Ssb2 upregulation in Rosa26-CreERT2 : Ssb1−/− mice. (A) PCR genotyping after tamoxifen induced Cre recombination mediated Ssb1 gene deletion. PCR analysis of recombination of the floxed Ssb1 allele in heterozygous Ssb1-floxed-Rosa26-CreERT2 (Rosa26-CreERT2 : Ssb1fl/+) mice and homozygous Ssb1-floxed-Rosa26-CreERT2(Rosa26-CreERT2 : Ssb1fl/fl) mice was performed ten days after the final tamoxifen injection. The efficacy of gene interruption in indicated tissues is shown. The PCR products of floxed (fl), wild type (wt) and deletion (null) alleles of Ssb1 were detected as 482, 360 and 118-bp bands, respectively. (B) Western blot analysis of Ssb1 protein in tissue extracts from mice following Cre recombination. Ssb1 protein levels were analyzed in the indicated tissues ten days after the final tamoxifen injection by immunoblotting with an antibody specific for Ssb1 and ß-actin as a loading control. (C) Western blot analysis of Ssb1 and Ssb2 protein in indicated tissues prepared from Rosa26-CreERT2 : Ssb1+/− and Rosa26-CreERT2 : Ssb1−/− mice ten days after Cre recombination. Ssb1+/− and Ssb1−/− mice were subjected to 6 Gy of total body irradiation (TBI). Indicated tissues were extracted 6 h post irradiation, and Ssb1 and Ssb2 protein levels were analyzed by immunoblotting. Immunoblotting of ß-Actin was used as a loading control. (TIF) [file pgen.1003298.s008.tif]

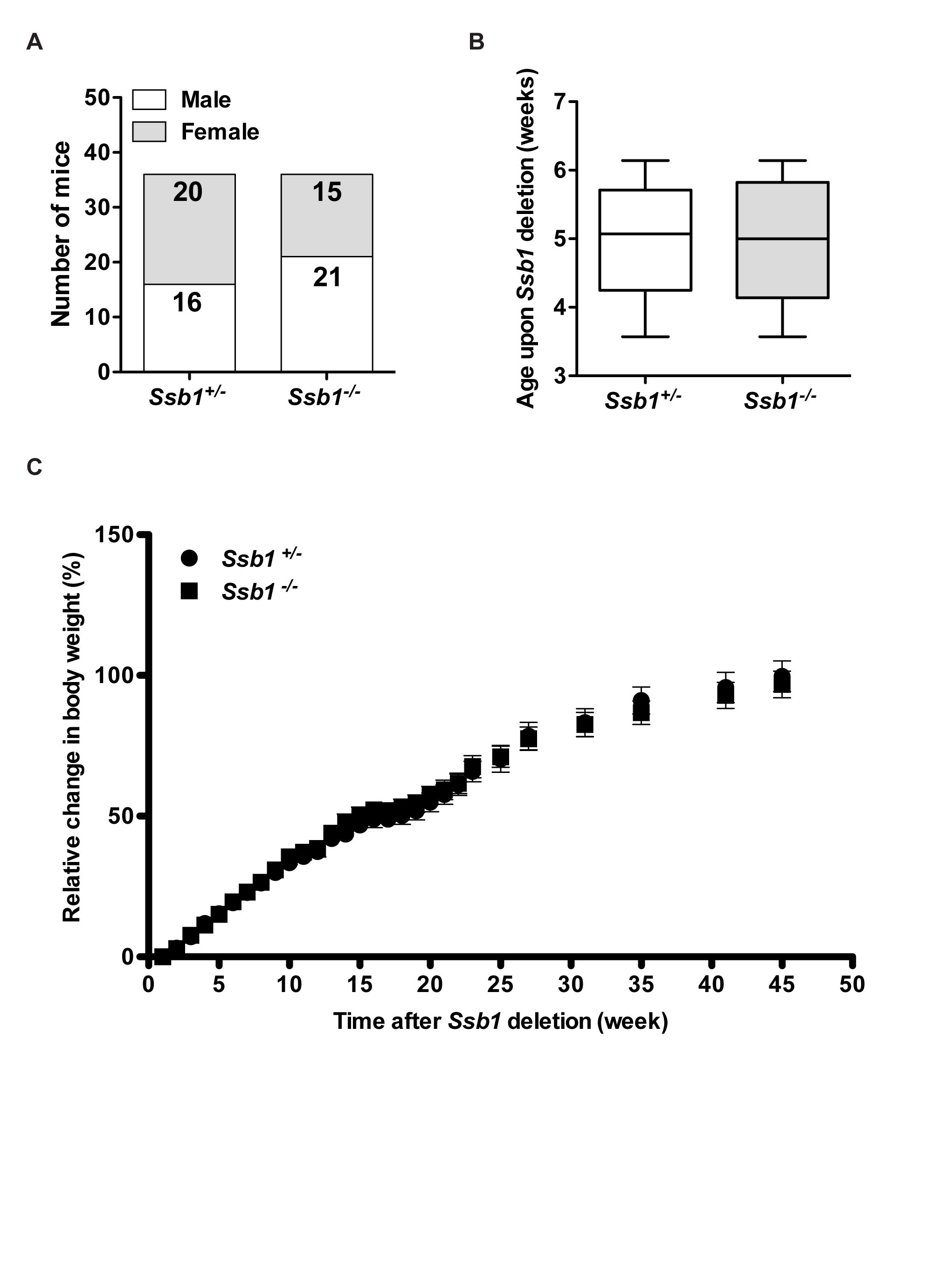

Supplement: Figure S9 — Comparison of body weights of Rosa26-CreERT2 : Ssb1+/− and Rosa26-CreERT2 Ssb1−/− mice. (A) Gender distribution comparison of Rosa26-CreERT2 : Ssb1+/−and Rosa26- CreERT2 : Ssb1−/− mouse cohorts. (B) Comparison of age of tamoxifen induction between cohorts. (C) Comparison of body weights of Rosa26-CreERT2 : Ssb1+/− and Rosa26-CreERT2 Ssb1−/− mice after tamoxifen injection (n = 35). (TIF) [file pgen.1003298.s009.tif]

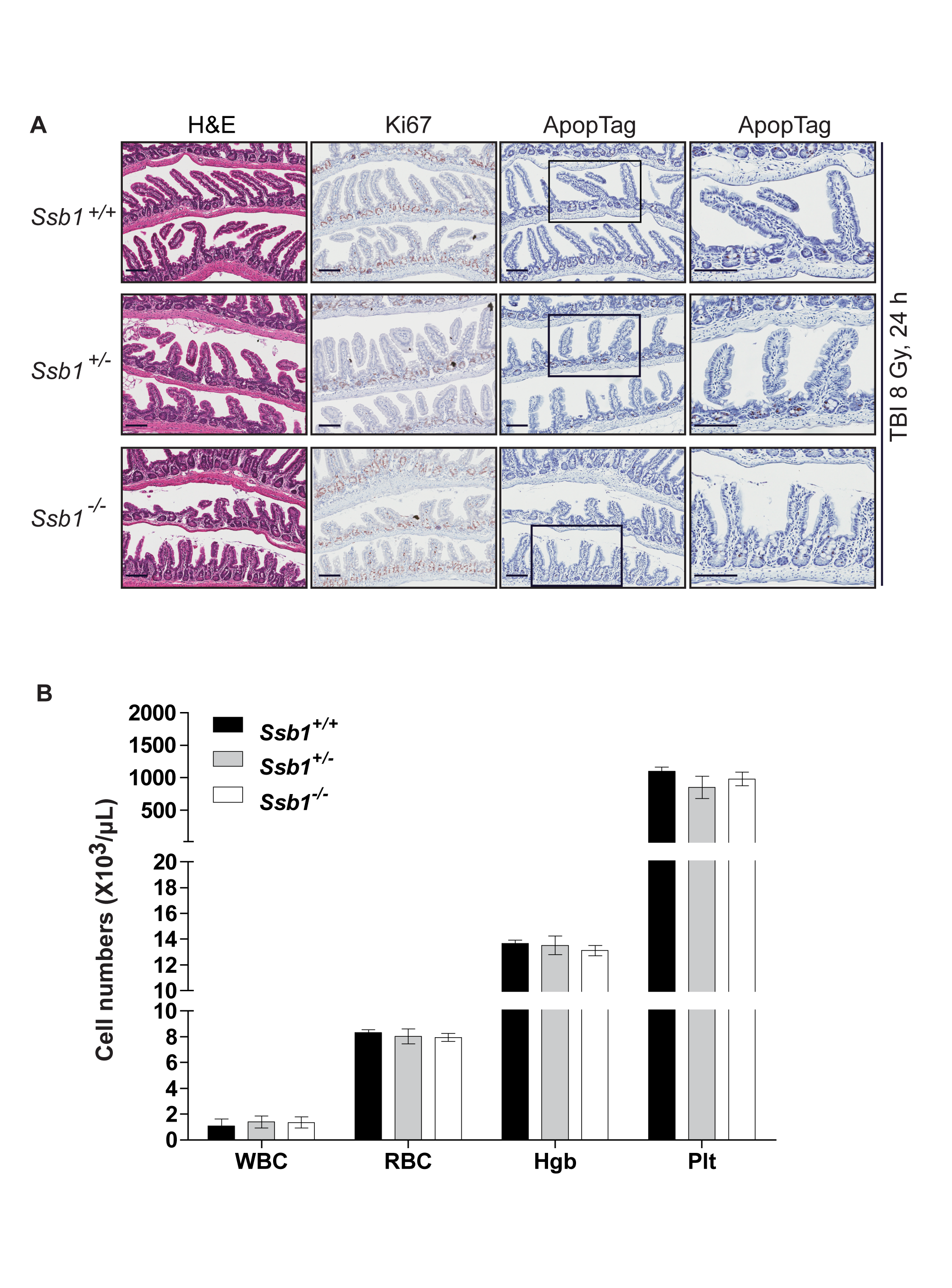

Supplement: Figure S10 — Histological analysis and complete blood count of mice at 24 h post total body irradiation (TBI). (A) Representative images of Haematoxylin and eosin, Ki67 (cell proliferation) and ApopTag (cell death) staining on small intestine sections from mice at 24 h post 8 Gy of TBI. (B) Complete blood count (CBC) analysis on peripheral blood from mice at 24 h post 8 Gy of TBI. Whole blood samples were processed for counts using Beckman Coulter ACT whole blood counter. Numbers of white blood cell (WBC), red blood cell (RBC), hemoglobin (Hgb), and platelets (Plt) were assessed. Scale = 100 µm. (TIF) [file pgen.1003298.s010.tif]

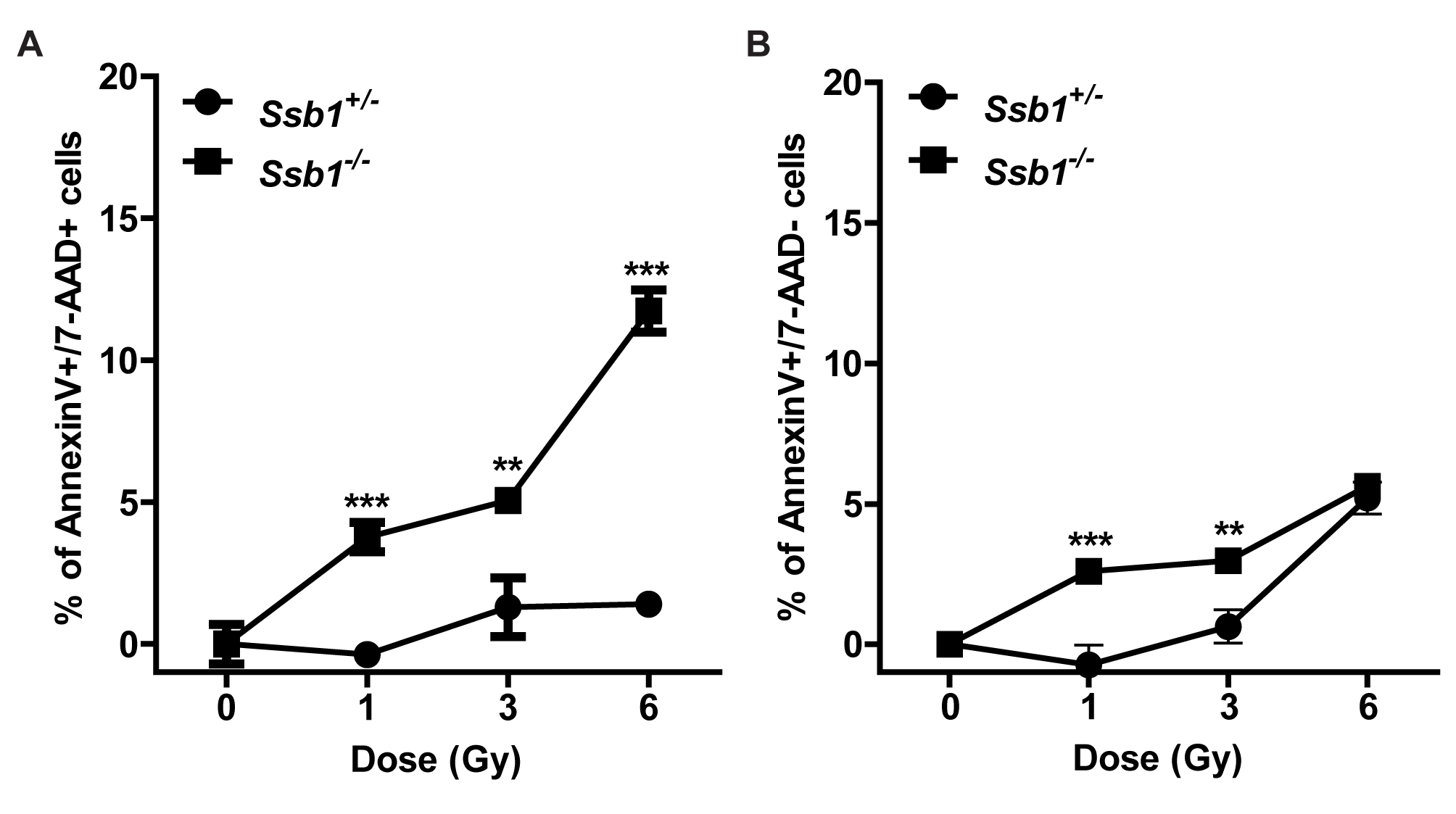

Supplement: Figure S11 — Assessment of radiosensitivity of thymocytes from Rosa26-CreERT2 : Ssb1+/− and Rosa26-CreERT2 : Ssb1−/− mice. Thymocytes were isolated from mice with indicated genotype and exposed to 1, 3 and 6 Gy of irradiation. (A) Percentage of cell death (Annexin V+/7-AAD+) of Ssb1+/− and Ssb1−/− thymocytes at indicted doses of irradiation (n = 3, **P<0.01, ***P<0.001; student's t-test). (B) Percentage of apoptotic cells (Annexin V+/7-AAD-) at indicated conditions (n = 3, **P<0.01, ***P<0.001; student's t-test). (TIF) [file pgen.1003298.s011.tif]

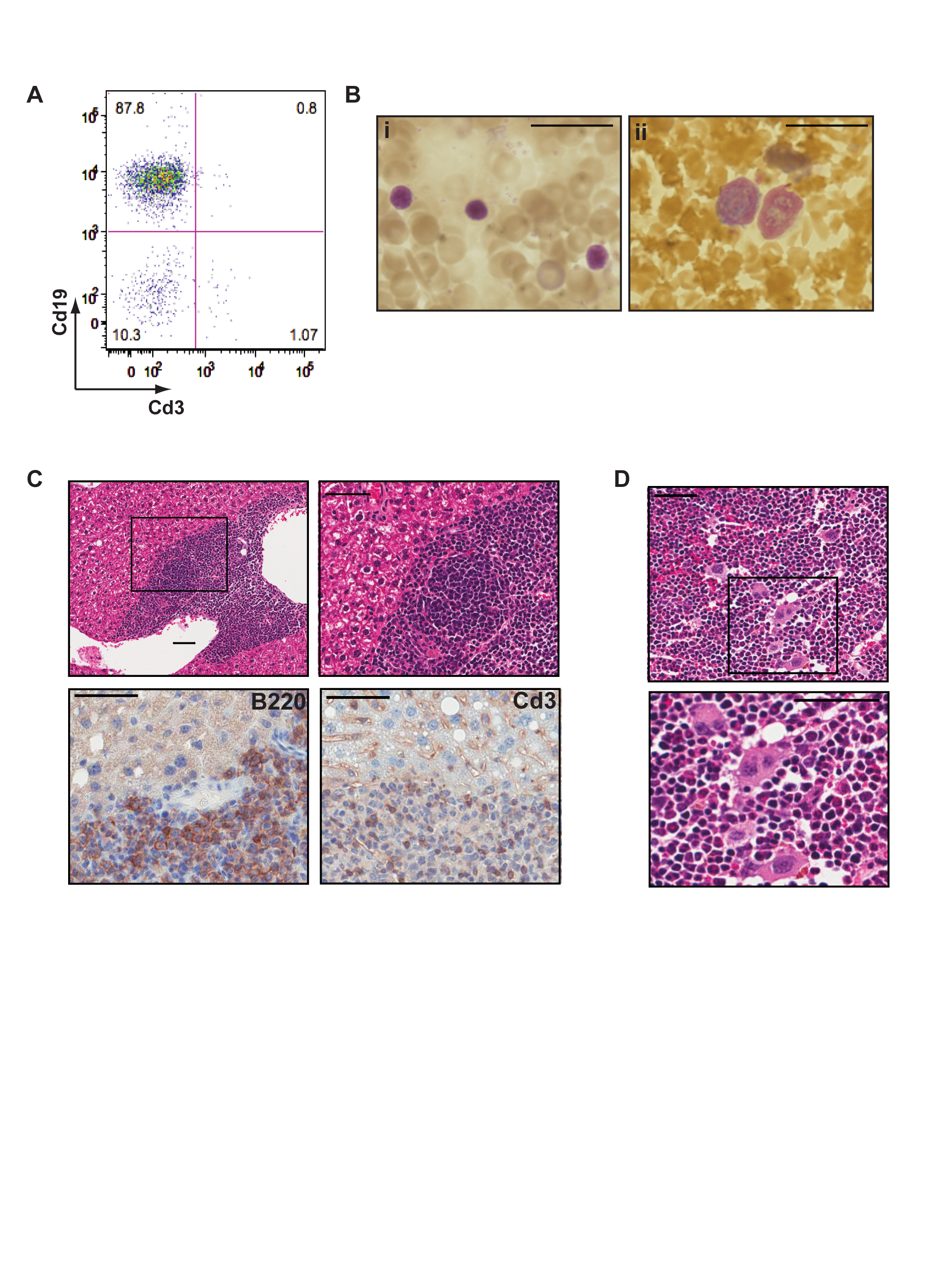

Supplement: Figure S12 — B-cell leukemia identified in a Rosa26-CreERT2 : Ssb1−/− mouse. (A) Representative flow cytometric analysis on lymphoblasts from peripheral blood (PB). Lymphoblasts were stained as Cd19 (B cell) positive lymphomas. (B) Wright's stain on PB smears showing lymphoblast cluster from a Rosa26-CreERT2 : Ssb1−/− mouse (ii) compared with a healthy control littermate (i). Leukocytes featured as large-sized undifferentiated haematopoietic cells with a small basophilic cytoplasm and visible nucleoli (Scale = 20 µm). (C) Lymphocytic leukemia involving the liver. Representative images of Haematoxylin and eosin stained sections (upper panel) and immunohistochemical staining of B220 or Cd3 (lower panel) showing periportal infiltration by B-lymphocytes (Scale = 50 µm). (D) Effacement of the lymph node architecture. Low-power (upper panel) and enlarged views (lower panel) show a periportal lymphocytic infiltrate in the lymph node (Scale = 50 µm). (TIF) [file pgen.1003298.s012.tif]

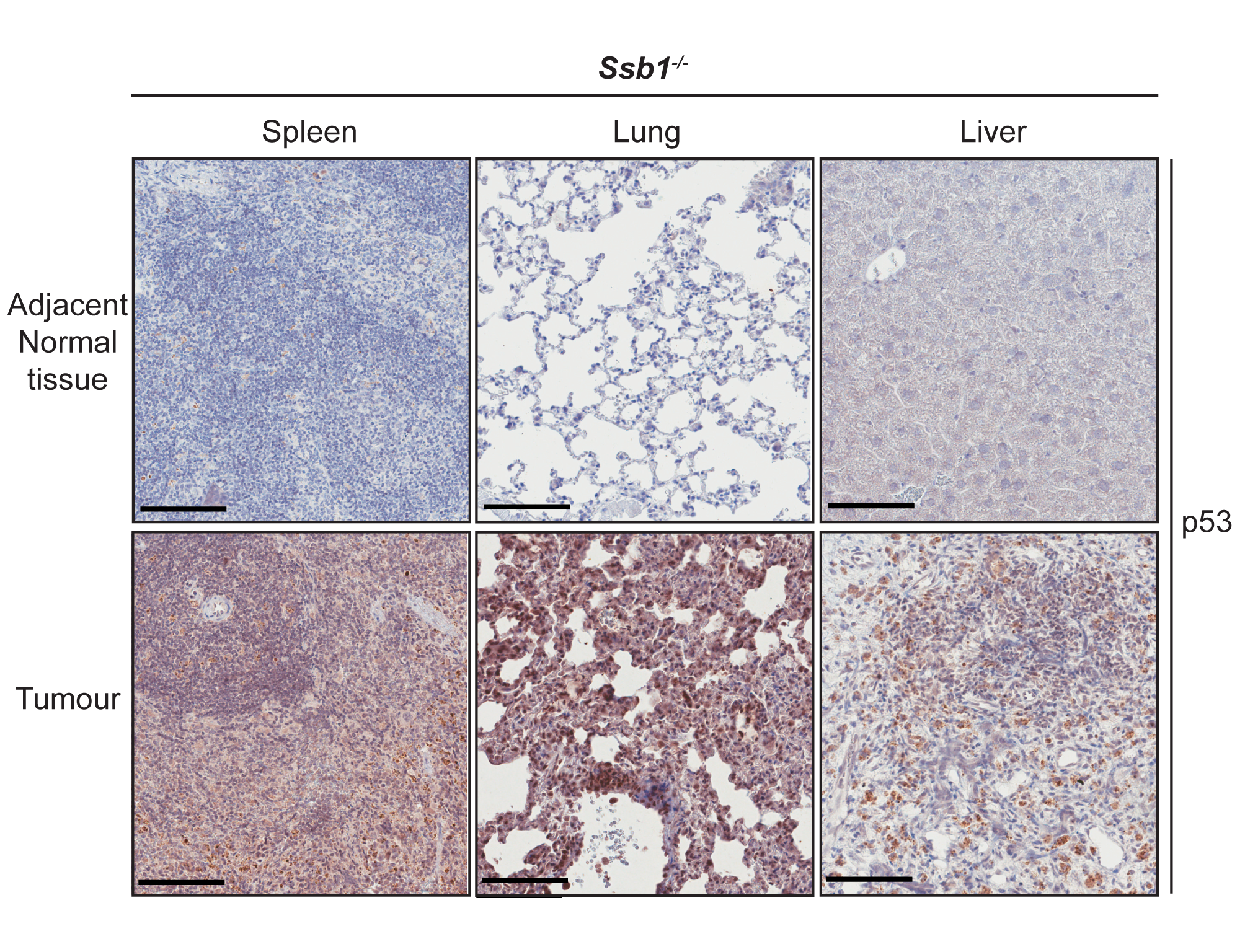

Supplement: Figure S13 — Representative images of p53 immunohistochemistry staining on tumour sections from Rosa26-CreERT2 : Ssb1−/− mice. Tumours developed in indicated organs from Rosa26-CreERT2: Ssb1 −/− mice were stained with p53 antibody (bottom panel) and compared with adjacent normal tissue from the same mice (top panel), Scale = 100 µm. (TIF) [file pgen.1003298.s013.tif]

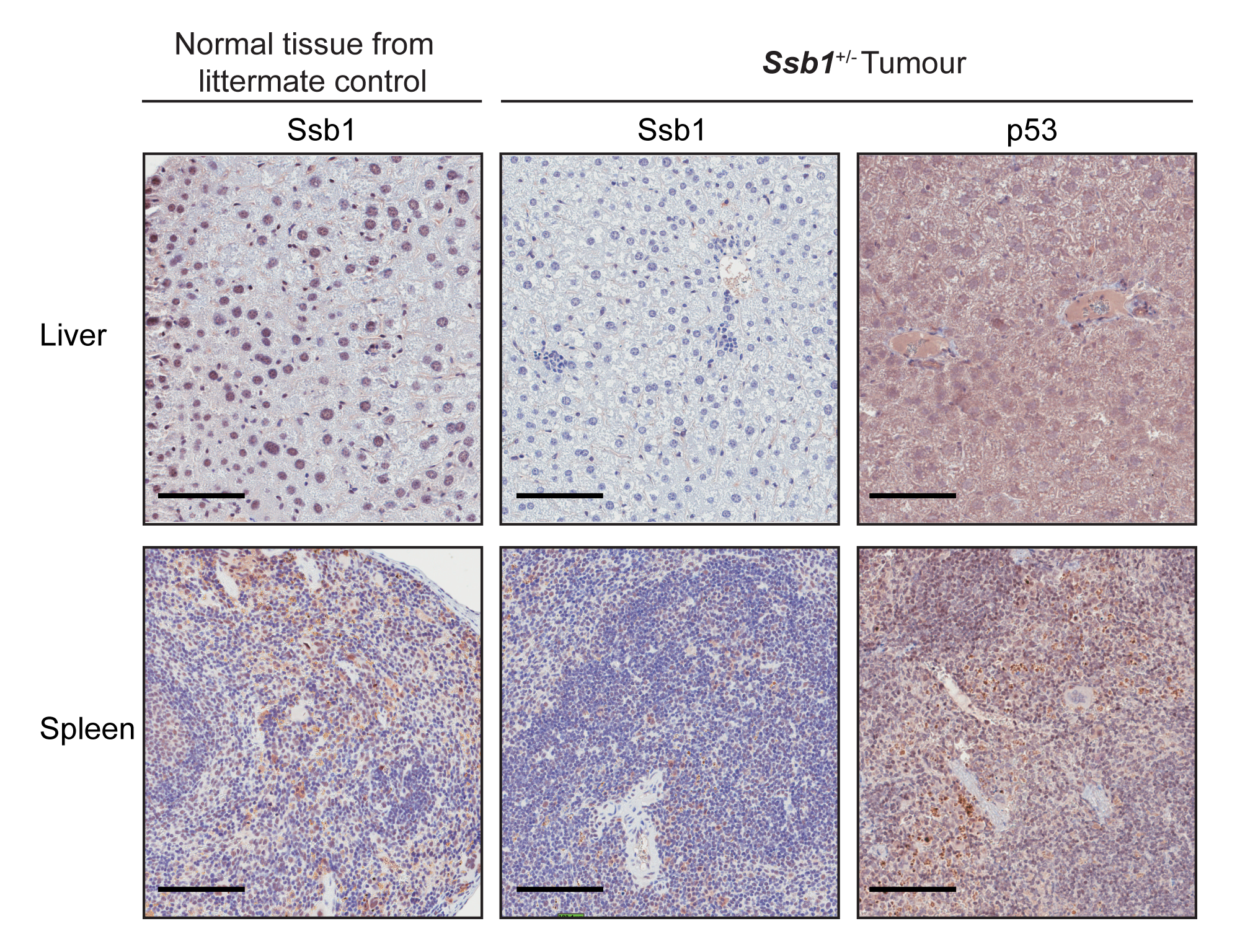

Supplement: Figure S14 — Representative images of Ssb1 and p53 immunohistochemistry staining on tumour sections from Rosa26-CreERT2 : Ssb+/− mice. Two tumours observed in Rosa26-CreERT2: Ssb1 +/− mice were stained with Ssb1 and p53 antibodies. Left panel is the control staining of Ssb1 on the respective organs from littermate control of Rosa26-CreERT2: Ssb1 +/− mice. Middle panel is Ssb1 staining from the Rosa26-CreERT2: Ssb1 +/− mice which developed tumours. Right panel is p53 staining of the tumour sections. Scale = 100 µm. (TIF) [file pgen.1003298.s014.tif]
